# The Relationship between Fungal Diversity and Invasibility of a Foliar Niche—The Case of Ash Dieback

**DOI:** 10.3390/jof6030150

**Published:** 2020-08-26

**Authors:** Ahto Agan, Rein Drenkhan, Kalev Adamson, Leho Tedersoo, Halvor Solheim, Isabella Børja, Iryna Matsiakh, Volkmar Timmermann, Nina Elisabeth Nagy, Ari Mikko Hietala

**Affiliations:** 1 Natural History Museum and Institute of Ecology and Earth Sciences, University of Tartu, 50411 Tartu, Estonia; leho.tedersoo@ut.ee; 2 Institute of Forestry and Rural Engineering, Estonian University of Life Sciences, 51006 Tartu, Estonia; rein.drenkhan@emu.ee (R.D.); kalev.adamson@emu.ee (K.A.); 3 Norwegian Institute of Bioeconomy Research, p.b. 115, 1431 Ås, Norway; halvor.solheim@nibio.no (H.S.); isabella.borja@nibio.no (I.B.); volkmar.timmermann@nibio.no (V.T.); nina.nagy@nibio.no (N.E.N.); 4 Institute of Forestry and Park Gardening, Ukrainian National Forestry University, 79057 Lviv, Ukraine; iramatsah@ukr.net; 5 Norwegian Institute of Bioeconomy Research, p.b. 2609, 7734 Steinkjer, Norway; ari.hietala@nibio.no

**Keywords:** *Hymenoscyphus fraxineus*, *Venturia fraxini*, mycobiome, epiphytic and endophytic fungi, qPCR, PacBio

## Abstract

European ash (*Fraxinus excelsior*) is threatened by the invasive ascomycete *Hymenoscyphus fraxineus* originating from Asia. Ash leaf tissues serve as a route for shoot infection but also as a sporulation substrate for this pathogen. Knowledge of the leaf niche partitioning by indigenous fungi and *H. fraxineus* is needed to understand the fungal community receptiveness to the invasion. We subjected DNA extracted from unwashed and washed leaflets of healthy and diseased European ash to PacBio sequencing of the fungal *ITS1-5.8S-ITS2* rDNA region. Leaflets from co-inhabiting rowan trees (*Sorbus aucuparia*) served as a reference. The overlap in leaflet mycobiomes between ash and rowan was remarkably high, but unlike in rowan, in ash leaflets the sequence read proportion, and the qPCR-based DNA amount estimates of *H. fraxineus* increased vigorously towards autumn, concomitant with a significant decline in overall fungal richness. The niche of ash and rowan leaves was dominated by epiphytic propagules (*Vishniacozyma* yeasts, the dimorphic fungus *Aureobasidion pullulans* and the dematiaceous hyphomycete *Cladosporium ramotenellum* and *H. fraxineus*), and endophytic thalli of biotrophs (*Phyllactinia* and *Taphrina species*), the indigenous necrotroph *Venturia fraxini* and *H. fraxineus*. Mycobiome comparison between healthy and symptomatic European ash leaflets revealed no significant differences in relative abundance of *H. fraxineus*, but *A. pullulans* was more prevalent in symptomatic trees. The impacts of host specificity, spatiotemporal niche partitioning, species carbon utilization profiles and life cycle traits are discussed to understand the ecological success of *H. fraxineus* in Europe. Further, the inherent limitations of different experimental approaches in the profiling of foliicolous fungi are addressed.

## 1. Introduction

Globalization has created an unprecedented movement of species across continents. These concern also fungi that can hitchhike along with imported seed, plants, soil (potted plants) or plant material like timber [1]. Some of the imported fungi that have pathogenic potential can cause huge ecological and economic damage on evolutionary naïve plants that, due to lack of co-evolution with the incomer, have no effective defense mechanisms against it. Classic examples of pandemics caused by invasive alien tree pathogens include Dutch elm disease caused by *Ophiostoma ulmi* (Buisman) Melin and Nannf. and *O. novo-ulmi* Brasier, and chestnut blight caused *Cryphonectria parasitica* (Murrill) Barr, diseases that initiated during early 20th century and decimated billions of elm and chestnut trees in North America and Europe [2,3].

Conventionally, in the frame of the disease triangle [4], the focus of the scientific community engaged with tree pandemics, and plant diseases in general, has been to decipher how host defense responses and developmental phases, pathogen biology (life cycle, pathogenicity factors) and abiotic factors, like weather influence the outcome of the tree–pathogen interaction. However, an emerging view is that microorganisms critically affect host physiology and performance, suggesting that the evolution and ecology of plants and animals can only be understood in a holobiont (host and its associated microorganisms) context [5]. The host microbiome is hypothesized to extend the phenotype of the host organism [6]. Besides this role, the fungi associated with plants often occupy critical roles in carbon and nutrient cycling of terrestrial ecosystems as well [7].

Concerning invasive alien plant pathogens and their interaction with the native fungal community, the ‘Diversity Resistance’ hypothesis argues that species rich communities should be highly competitive and readily resist invasion [8]. The underlying assumption is that niche space in diverse natural communities is a limiting factor, and that such communities are structured by interspecific competition [6,8]. Several observational and/or experimental studies have suggested that local biodiversity [9], the presence and absence of niche specialists and generalists, and the type and number of incoming colonists into the recipient ecosystem [10], influence the outcome of species introductions. Additional factors that can influence the local establishment and subsequent invasive phase of an introduced species include propagule pressure [11], the similarity of recipient community structure at different locations [12], the viability of small introduced populations [13], and the presence of specific ecological drivers [14].

Dieback of European ash represents the most recent pandemic in trees that is currently threatening the tree species at a continental scale in Europe. The disease agent *Hymenoscyphus fraxineus* (T. Kowalski) Baral, Queloz and Hosoya [15] (syn. *H. pseudoalbidus* V. Queloz, C.R. Grünig, R. Berndt, T. Kowalski, T.N. Sieber and O. Holdenrieder [16], Helotiales, Ascomycetes) is a relatively recently discovered pathogen in Europe [15,17]. The disease, first observed in Poland in the early 1990s [18], has spread rapidly across the continent [19,20]. Its main host, European ash, is growing naturally in mixed forests with other broadleaved trees over a wide range of altitudes and soil moisture conditions across Europe [21]. European ash is one of the most important pioneer tree species in western, central and northern regions of the continent, and is considered a principal tree species in the prospect of biological diversity and industrial use for furniture and construction [22]. Ash dieback has emerged as one of the most serious problems in European forests, as the disease is threatening this keystone tree species at a continental scale [22]. Thus, *H. fraxineus* is listed as one of the most serious invasive forest disease agents [23], accompanied with huge economical losses as well; the total cost of the dieback of European ash is estimated at £15 billion for Britain alone [24].

The pathogen probably originates from East Asia, where it is associated with leaves of native Asian ash species [25,26,27,28,29,30,31], and causes only minor shoot dieback symptoms in the Russian Far East [29]. In Europe, besides European ash, this invasive fungus causes shoot dieback in the native narrow-leafed ash *F. angustifolia* Vahl. [31,32], which is still unaffected in its southern range. The pathogen is also able to colonize leaves, and to a varying extent, shoot tissues of the North American ash species *F. nigra* Marsh., *F. pennsylvanica* Marsh. and *F. americana* L., and the Asian ash species *F. mandshurica* Rupr., *F. chinensis* Roxb. and *F. Sogdiana* Bunge growing in European parks and arboreta [30,32,33,34,35]. The infected ash trees die eventually, although the progress of the disease is much slower in older trees [36,37,38]. Importantly, there are obvious genetic differences between different individuals of European ash in the susceptibility to shoot infection by *H. fraxineus*—field observations suggest that ~1%–5% of the European ash trees possess tolerance against this fungus [19].

Invasive pathogens may deteriorate the health of evolutionary naïve host populations that have not co-evolved with the pathogen. The success of invaders depends also on their traits relative to those of the native species competing for the same niche [39]. Concerning *H. fraxineus*, the traits of indigenous species associated with ash leaves are the most relevant, as leaf infection serves apparently as a route to shoot colonization by this pathogen [40]. Moreover, the pathogen forms its fruiting bodies on petioles, rachis and leaflet vein tissues of the compound ash leaf on the forest floor in the season following leaf shed. The mycobiome of healthy ash leaves is formed by basidiomycetous yeasts with low host specificity, a few highly-specific biotrophic fungi, and numerous endophytes or facultative parasites, which may have competence for endophytic overwintering in buds and/or saprobic overwintering in ash leaf petioles [41]. *Hymenoscyphus fraxineus* has been considered to be specific for *Fraxinus* spp., while most of the endophytic and epiphytic species associated with foliage of angiosperm trees are shared among many species [42,43,44]. The level of richness and the degree of functional complementarity among indigenous fungi associated with ash leaves are high [44,45,46]. At face value the niche would appear to host a competitive community in comparison to, for example elm xylem, the primary niche of the causative fungus of Dutch elm disease [47]. No studies are available regarding the fungal species richness in the inner bark for chestnut, the primary niche of *C. parasitica*, but in general, inner bark appears to host highly structured fungal communities, with a large proportion of tree-specific fungal species [48]. Resource competition theory predicts that multiple species can coexist if species have trade-offs in their traits and if the habitat is spatially or temporally heterogeneous [39]. This prediction applies fully to leaves of deciduous trees that host few fungi directly after flushing but undergo pronounced changes in fungal community size and structure during the course of the growing season [7].

It is reasonable to presume that interactions take place between propagules of the indigenous mycobiome and *H. fraxineus* during the pre-penetration phase on leaf surface and during tissue entry and infection, but the experimental design of prior studies on ash leaf mycobiomes [44,45,46,49,50] did not allow differentiation between epiphytic and endophytic propagules, a main motivation of the current study.

To further the understanding of the pathogen and fungal community specific traits that facilitate the ecological success of *H. fraxineus* in Europe, we have now profiled the seasonal changes in leaflet mycobiomes of ash tree genotypes that differ in their phenotypic response to *H. fraxineus*. The study was conducted at one site in Estonia and one site in Norway in order to take into consideration any local variation in ash leaflet mycobiome. In Estonia, the pathogen has been broadly distributed all over the country since 1997 [51]. In Norway, ash dieback has been present since 2006, and the disease has now spread all over the natural range of European ash in Norway [20], including Central Norway, which harbours the northernmost ash forests in Europe. As a proxy, we also monitored the leaflet mycobiome of a co-inhabiting non-host tree species, rowan (syn. mountain ash, *Sorbus aucuparia* L.), which is not challenged by any epidemic disease. While phylogenetically unrelated, European ash and rowan are widespread in Europe, co-occur commonly at the same forest site, tolerate shade when young, and possess pinnate compound leaves and superficially similar leaf buds, which may render their comparison more relevant than comparisons with other species. We postulated the following research questions: do the pools of epiphytic and endophytic propagules of indigenous fungi associated with leaflets of co-inhabiting rowan and European ash trees, with or without shoot dieback symptoms, show differences in composition and seasonal trajectory? Can eventual differences be related to *H. fraxineus*? To answer these questions, we sampled European ash and rowan leaves in Estonia and Norway throughout the vegetation period of 2014, and subjected DNA from washed and unwashed leaflets to fungal DNA sequencing, community analysis, and qPCR profiling of *H. fraxineus* propagule level.

## 2. Materials and Methods

### 2.1. Study Sites and Sampling

Sampling was carried out during the vegetation period of 2014 in two sampling sites: Ås, Norway (59°40′44′′ N, 10°46′31′′ E, 100 m a.s.l.) and Vedu, Estonia (58°29′06.4′′ N 26°45′25.2′′ E, 100 m a.s.l). The Norwegian site, a naturally regenerated forest, is a moist stand with rich understory vegetation dominated by meadowsweet (*Filipendula ulmaria* (L.) Maxim.). European ash is present both as a canopy forming tree (the largest ash trees ranging between 20 and 27 m in height) and in the understory along with rowan, aspen (*Populus tremula* L.), bird cherry (*Prunus padus* L.), downy birch (*Betula pubescens* Ehrh.), alder (*Alnus* spp.) and willows (*Salix* spp.). The average height of sampled ash and rowan trees was 3 and 4 m, respectively. The Vedu site, situated in abandoned agricultural land, includes *F. excelsior* trees in all age classes up to 70 years old. The overstory is dominated by ash, followed by common oak (*Quercus robur* L.) and Norway maple (*Acer platanoides* L.) at 85%, 10% and 5% frequency, respectively. The understory is dominated by ash, rowan, Norway maple, some old apple trees (*Malus domestica* Borkh. nom. illeg.) and currants (*Ribes* spp.). The height of sampled European ash and rowan trees ranged between 4–15 and 2–8 m, respectively. Samples were taken approximately 2–3 m above ground.

At both sites, leaves were collected at 1–4 week intervals across the season from selected and marked trees within each of the following groups: (1) two ash trees showing obvious signs of *H. fraxineus* infection in their shoots, (2) two ash trees without any shoot symptoms, and (3) two rowan trees. One compound leaf for each ash and rowan tree was sampled per time point. Two randomly chosen leaflet pairs per compound leaf were processed. For each sampling time, 12 Estonian samples and 12 Norwegian samples were subjected to fungal DNA sequencing, totaling 24 (four samples from healthy ash, four from diseased ash and four from rowan subjected to washing and the same number of samples (4 + 4 + 4) processed without washing). Samples from three sampling times were subjected to DNA sequencing, this totaling 72 (24 + 24 + 24) samples per site, altogether 144 samples (see Supplementary Materials Figure S1).

In Estonia, an approximately 1 cm^2^ subsample was taken randomly from each leaflet of chosen leaflet pairs so that the sampled blade area included segments of 1–2 side veins (see Supplementary Materials Figure S2). In Norway the whole leaflets of randomly chosen leaflet pairs were processed. In each sampling scheme—for normalization purposes—the samples were weighed prior to processing. To remove propagules residing on the tissue surface, one leaflet sample was washed in Tween 20 detergent (one drop of Tween per 500 mL of distilled water) for one hour while shaking (50 rpm) prior to pulverizing and DNA extraction, and the other leaflet sample from the same leaflet pair was processed without washing.

Real-time PCR profiling of *H. fraxineus* DNA level was carried out for the sampled leaflets (Norway, Estonia) and air samples, collected across the season by solar power-driven Burkard 7-d volumetric spore samplers (Burkard Scientific, Uxbridge, UK) installed at ground level and 5 m above ground at the subjected stand (Norway). Based on these data, we identified three time-points that corresponded either to the onset of *H. fraxineus* sporulation (June), the peak of sporulation (late July/early August) or low-level late season sporulation with necrotic symptoms visible on leaf tissues (September; Supplementary Materials Figure S3).

### 2.2. Leaflet Inoculation Experiment

To obtain reference data for the behavior of *H. fraxineus* ascospores on ash leaf surface, an artificial inoculation of detached leaflets was set up. For this purpose, compound leaves were collected from 3–4 m tall European ash saplings from the municipality of Stjørdal, Central Norway, in August 2014. At this time, the region was still free of ash dieback. One day after the leaf collection, randomly chosen leaflets were subjected to deposition of *H. fraxineus* ascospores in laboratory conditions at Ås, southeastern Norway. A petiole segment with a fresh *H. fraxineus* ascocarp, collected from the same site as the leaves subjected to mycobiome analysis, was taped onto the inner side of a Petri dish cover, so that the hymenium was facing down. A leaflet rinsed in sterile distilled water was placed under the ascocarp, the abaxial side facing the ascocarp. The petiole segment with ascocarp was removed after an incubation time of 12 h. Leaflets were collected 34, 48 and 96 h after the start of the experiment and processed by using a modification of the method of Koske and Gemma [52]. This involved tissue clearing by heating at 90 °C for 30 min in 2.5% KOH, followed by acidification in 1% HCl at room temperature for 24 h and staining in acidic glycerol (500 mL glycerol, 450 mL H_2_O, 1% HCl) containing 0.05% trypan blue at 90 °C for 10 min. For destaining, the leaflets were placed in acidic glycerol prior to storage in glycerol at room temperature in darkness. For examination under a light microscope, the destained leaflets were placed under a cover slip in a Petri dish.

### 2.3. Molecular Analysis

For the samples from Estonia, DNA extraction from the entire leaflet subsample was carried out using GeneJET Genomic DNA purification kit (Thermo Fischer Scientific, Vilnius, Lithuania) according to Drenkhan et al. [30]. For the samples from Norway, DNA extraction was carried out using 20 mg pulverised leaflet tissue and Qiagen DNeasy Plant Mini Kit according to manufacturer’s instructions (Qiagen, Hilden, Germany). In both cases, primers ITS4ngs [53] and ITS1catta [54] were used to amplify fungal DNA. The PCR products were sequenced using PacBio platform in the University of Oslo in Norway. PacBio has recently been successfully used in metabarcoding analysis of microorganisms, as the long DNA barcodes of 500–1500 bp improve species identification [54,55,56]. The primer ITS1catta was originally designed specifically for this study in order to differentiate *Hymenoschyphus albidus* (Roberge ex Desm.) W. Phillips from *H. fraxineus*, while excluding amplification of plant DNA and to avoid the long intron in the 3′ end of the rRNA 18S gene of *Hymenoscyphus* species (for tests of performance, see also [55]) a feature of that particular primer that has not been demonstrated before. The ITS4ngs primer was equipped with a 10–12 base multiplex identifier (MID) index that differed from any other 107 indices by at least four bases.

Conventional PCR was carried out with two replicates for each sample in 25 µL reaction volume containing 0.5 μL of forward and reverse primer and 5 μL of HOT FIREPol Blend Master Mix Ready to Load (Solis BioDyne, Tartu, Estonia). Amplification was performed as follows: 15 min at 95 °C, followed by 25 cycles of 30 s at 95 °C, 30 s at 55 °C, 1 min at 72 °C, and a final step at 72 °C for 10 min. The PCR reactions were checked for the presence of a product on 1% agarose gels. In case of no visible band, we repeated the amplification by increasing the number of cycles up to 35. The PCR products were purified using GeneJet DNA purification kit (Thermo Fischer Scientific, Vilnius, Lithuania) following the manufacturer’s instructions.

The amplicons were pooled into two sequencing libraries (separately for two sites) on equimolar basis. Library preparation followed the protocols established for the RSII instrument of PacBio third-generation sequencing platform (Pacific Biosciences, Inc. Menlo Park, CA, USA). The libraries were loaded to SMRT cells using the diffusion method. Sequencing was performed using P6-C4 chemistry for 10 h following Tedersoo et al. [57].

Quantitative PCR (qPCR) was carried out in 20-μL-reaction volumes using the *H. fraxineus* specific assay designed by Ioos et al. [58]. For the Estonian samples, the reaction mix included 1 μL of fluorescent tag and 4 μL of x HOT FIREPol Blend Master Mix Ready to Load (Solis BioDyne, Tartu, Estonia). Amplification was performed according to Ioos et al. [58] with some modifications related to the PCR mixture: an initial denaturation at 95 °C for 15 min, followed by 40 cycles of denaturation at 95 °C for 15 s, and primer binding in 60 °C for 55 s using Rotor-Gene Q MDx qPCR machine (Qiagen, Hilden, Germany). For the Norwegian samples, Takyon™ Low Rox Probe MasterMix dTTP Blue (Eurogentech, Seraing, Belgium) was used according to manufacturer instructions with Applied Biosystems ViiA 7 qPCR (Thermo Fischer Scientific, Vilnius, Lithuania) machine and the above described cycling parameters, except that 65 °C was used at the annealing and extension phases. Standard curves for DNA quantity were constructed with the PCR conditions used in each country, based on DNA extracted from pure cultures of *H. fraxineus*. The obtained Ct values were plotted against log-transformed template DNA amounts to prepare a standard curve to quantify pathogen DNA by interpolation in leaflet samples.

### 2.4. Bioinformatics Analysis

Bioinformatics was carried out by using various programs implemented in Pipecraft v1.0 [59]. Using mothur (v1.36.1) [60], reads < 100 bp were removed and longer sequences were demultiplexed allowing 1-base differences to index and 2-base differences to primer. Using UCHIME [61], de novo chimera filtering was performed. The full-length Internal Transcribed Spacer (ITS) region was extracted from the rRNA genes using ITSx (v1.0.11) [62]. Using CD-HIT (v4.6) [63], sequences were clustered into Operational Taxonomic Units (OTUs) based on 99% sequence similarity. As clustering may merge *H. fraxineus* and *H. albidus* sequences, we added one *H. albidus* sequence manually in order to evaluate this possibility. The remaining OTUs were taxonomically identified based on representative sequences against the UNITE v.7 database [64]. OTUs were considered as members of Fungi if their representative sequences matched best fungal taxa at *e*-value < *e*-50. Representative sequences that had >97% sequence similarity to reference sequences were assigned to species hypotheses (SHs) based on UNITE [64]. Higher level classification of Fungi was based on the e-value and sequence similarity criteria of Tedersoo et al. [53].

### 2.5. Statistical Analysis

OTU richness was calculated for each sample, using PAST v3.25 [65] for rarefaction to check if the number of samples was sufficient to capture most of the species diversity. The statistical calculations were done in R studio version 1.1.456 package lme4 [66], where sampling site was added as a random factor and square root of total number of sequences per sample served as a covariate. A possible effect of tree health, date of sampling and treatment on the abundance of *H. fraxineus* was tested using a linear mixed model. Calculations of differences in log-transformed qPCR estimates of *H. fraxineus* DNA level between ash phenotype, tree species, treatment and sampling date were done in Excel using ANOVA with Tukey HSD—differences with *p* value ≤ 0.05 were considered significant. Extrapolation of total fungal biomass using qPCR and read percentage data for *H. fraxineus* was performed according to Cross et al. [45]. We also compared PacBio sequence read percentages of detected species between unwashed and washed leaflets using ANOVA with Tukey HSD. Differences between ash phenotype, treatment and site were considered significant with *p* value ≤ 0.1.

To test for differences in fungal communities between the two sites, two tree species, two treatments (washed and unwashed) and two ash phenotypes (trees with shoot dieback symptoms vs. asymptomatic trees), we used PERMANOVA+ [67]. OTU abundance matrix was square-root transformed to reduce the effect of dominant species. Bray–Curtis dissimilarity [68] was used as a distance measure. Fungal community structure was visualized using PCoA as implemented in Primer v6 [69]. We also performed a probabilistic species co-occurrence analysis across all samples to detect any species that showed negative or positive association with *H. fraxineus*, using the R function co-occur [70]. These analyses were performed separately for both sites and tree species present at each site.

## 3. Results

### 3.1. qPCR Screening of Pathogen Sporulation and DNA Level in Leaflets

At both sites, *H. fraxineus* DNA amount on ash leaflets, based on qPCR screening, showed a vigorous increase towards autumn (Supplementary Materials Figure S3), whereas on rowan leaflets the pathogen DNA level remained generally low throughout the vegetation period. The qPCR analysis of airborne fungal propagules at the Norwegian site was consistent with pathogen DNA level profiles on ash leaflets; a low level of *H. fraxineus* propagules were detected in the second half of June, followed by a gradual increase in late July, peaking during the first half of August, and a rapid decline in late August (Supplementary Materials Figure S3). Based on this screening, we chose samples from 30th of June (both Estonia and Norway) to cover the early-season sporulation of *H. fraxineus*, 28th of July (Estonia) and 11th of August (Norway) to cover peak sporulation, and 1st of September (Norway) and 15th of September (Estonia) to cover late-season sporulation (Supplementary Materials Figure S3). The site-specific difference in the timing of 2nd and 3rd samplings was not intentional but due to different sample collection intervals in Estonia and Norway. No significant differences in *H. fraxineus* DNA level were observed between healthy and symptomatic ash trees in either country. In Norway, unwashed leaflets of ash showed significantly 3-fold (11th of August) to 44-fold (1st of September) higher *H. fraxineus* DNA amount than the corresponding washed leaflets, whereas no significant differences in *H. fraxineus* DNA amount were observed between unwashed and washed ash leaflets in Estonia.

For extrapolation of the total fungal DNA in the leaflets, we needed to have both the *H. fraxineus* qPCR estimate and its sequence read proportion for a sample—this criterion was met only for ash in September, since for several ash samples from the other months, and for rowan in general, *H. fraxineus* was not detected by both methods. In Estonia in September, the subsamples from unwashed leaflets of healthy and symptomatic ash trees had 0.43 and 0.33 ng fungal DNA per milligram tissue, respectively, and the respective amounts for washed subsamples were 0.007 and 0.005 ng. In Norway in September, the entire unwashed leaflets of healthy and symptomatic ash trees had 27 and 92 ng fungal DNA per milligram tissue, respectively, and the respective amounts for washed leaflets were 25 and 11 ng. Due to high sample specific variation, the differences between washing treatment or ash phenotype were not statistically significant (*p* > 0.05) in either country.

### 3.2. Factors that Influenced Fungal Community

**Figure 1:**
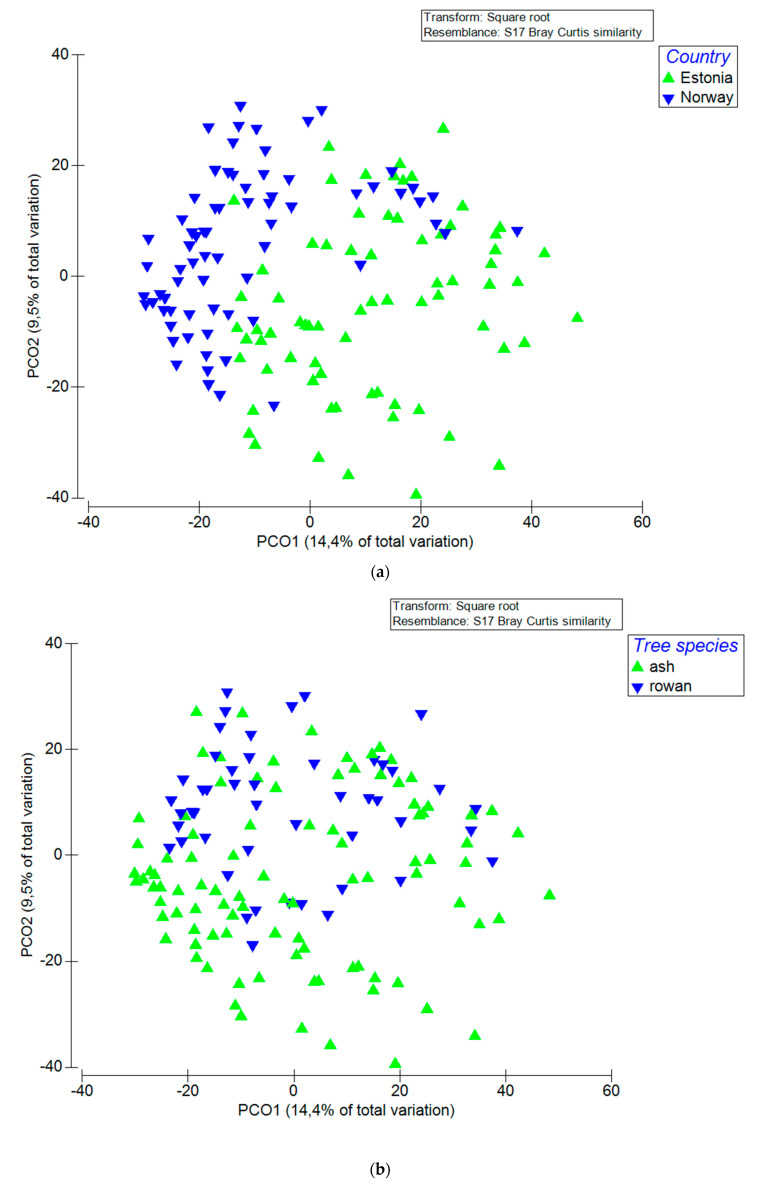

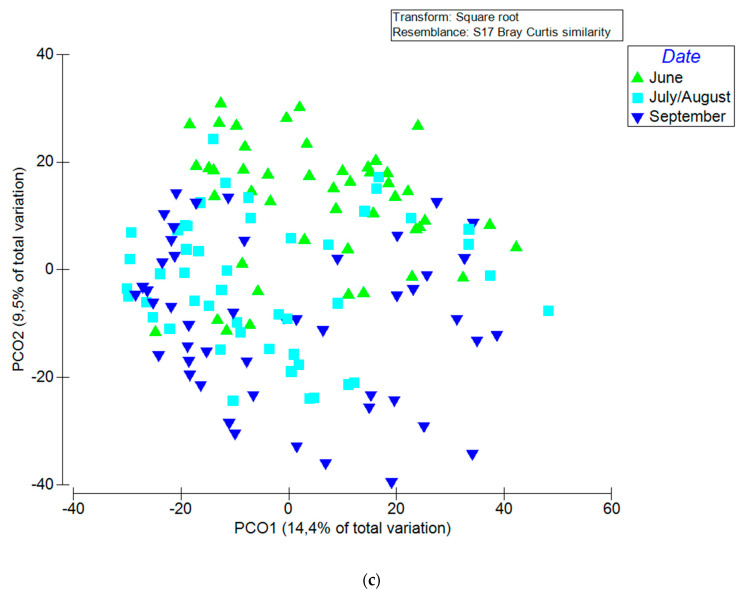
Principal coordinates analysis, presenting trends in fungal community structure for (**a**) the different sampling sites (countries), tree species (**b**) and sampling times (**c**).

Permutational ANOVA showed that the sampling site (country) was the most important factor, explaining 14.5% of the variation in fungal composition (*p* < 0.001), followed by the sampling time (4.9%; *p* < 0.001), occurrence of disease symptoms (3.7%; *p* < 0.001), washing treatment (2.1%; *p* < 0.005) and tree species (1.8%; *p* < 0.05). Interactions between tested factors also contributed to some variation: site x date, site x symptoms, date x symptoms as well as site x date x symptoms explained 2.6%, 3.3%, 1.5% and 1.8% of the variation, respectively, when data from both sites were pooled together. When considering the two sampling sites separately, in Norway the most important factor explaining variation in fungal composition was tree species (15.9%; *p* < 0.01), followed by sampling date (15.8%; *p* < 0.01), sampling date and symptom interaction (15.6%; *p* < 0.01), symptom (10.9%; *p* < 0.01), tree species and sampling date interaction (8.9%; *p* < 0.05). In Estonia, tree species explained the largest part of the variation (18.9%; *p* < 0.01), followed by sampling date x symptom interaction (14.7%; *p* < 0.01) and sampling date (14.5%; *p* < 0.01). The effects of sampling site, tree species and sampling date on fungal composition of pooled data are illustrated in the PCoA plots (Figure 1), the two primary axes collectively accounted for 23.9% of the variation in the fungal community. PCoA plots showing differences between different dates and tree species per site are included in (Supplementary Materials Figure S4).

### 3.3. Fungal Taxonomic Richness and Proportion of H. fraxineus

The total filtered sequence dataset contained 11,462 high-quality full-length ITS sequences from 144 samples (washed and unwashed), including 96 European ash and 48 rowan leaflets. In total, the data set consisted of 152 fungal OTUs, of which 63% were assigned to Ascomycota and 37% to Basidiomycota. Rarefaction analysis of sequences from unwashed and washed samples indicated that, on average, leaflets of European ash and rowan harboured 17.0 (±1.82) (mean ± SE) and 18.7 (± 2.20) (mean ± SE) fungal OTUs, respectively (F_1.68_ = 2.2; R^2^_adj_ = 0.658; *p* = 0.001). The tree species effect on species richness was significant for both unwashed (F_1.68_ = 0.6; R^2^_adj_ = 0.655; *p* < 0.001) and washed samples (F_1.68_ = 1.7; R^2^_adj_ = 0.655; *p* = 0.015). There was also a significant interaction between sites in both washed (F_1.68_ = 18.5; R^2^_adj_ = 0.655; *p* = 0.018) and unwashed (F_1.68_ = 8.9; R^2^_adj_ = 0.72; *p* = 0.048) samples. On average, the Norwegian site had 19.9 (±2.34) (mean ± SE) OTUs per sample and the Estonian site 15.2 (±1.79) (*p* < 0.05) (mean ± SE) OTUs per sample. The health status of ash did not affect fungal species richness on leaflets (F_1.90_ =2.6; R^2^_adj_ = 0.655; *p* = 0.300).

**Figure 2:**
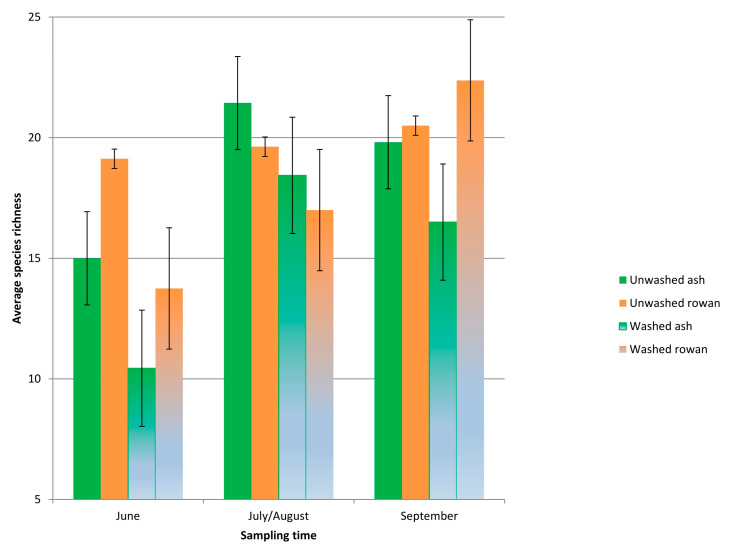
Average fungal species richness (species per sample) from PacBio data in unwashed and washed leaflets of ash and rowan across the sampling period. Data from Estonia and Norway pooled (*n* = 144).

Unwashed leaflets of rowan and ash showed generally higher fungal species richness than washed leaflets, except for rowan leaflets sampled in September (Figure 2), but the differences between treatments were not statistically significant. While the fungal species richness was higher in June and September on rowan leaflets than on ash leaflets, the opposite was observed in late July/early August. On rowan leaflets, fungal species richness showed a continuous increase across the season, but the differences between sampling times were not statistically significant. In contrast, on leaflets of European ash, fungal species richness had a steep increase towards July/August and then declined significantly in September in both unwashed and washed samples in comparison to the earlier sampling times (F_1.90_ = 21.0; R^2^_adj_ = 0.655; *p* < 0.0001; Figure 2). The decline in species richness coincided with an increase in biomass of *H. fraxineus*, estimated by qPCR (Figure S3). There was a strong linear correlation between *H. fraxineus* read percentage and qPCR estimates of *H. fraxineus* DNA level on ash leaflets (product moment correlation coefficient r^2^ = 0.82 for Norway and 0.64 for Estonia).

### 3.4. Host, Season and Treatment—Specific Patterns in Sequence Abundance of Fungal Species

**Figure 3:**
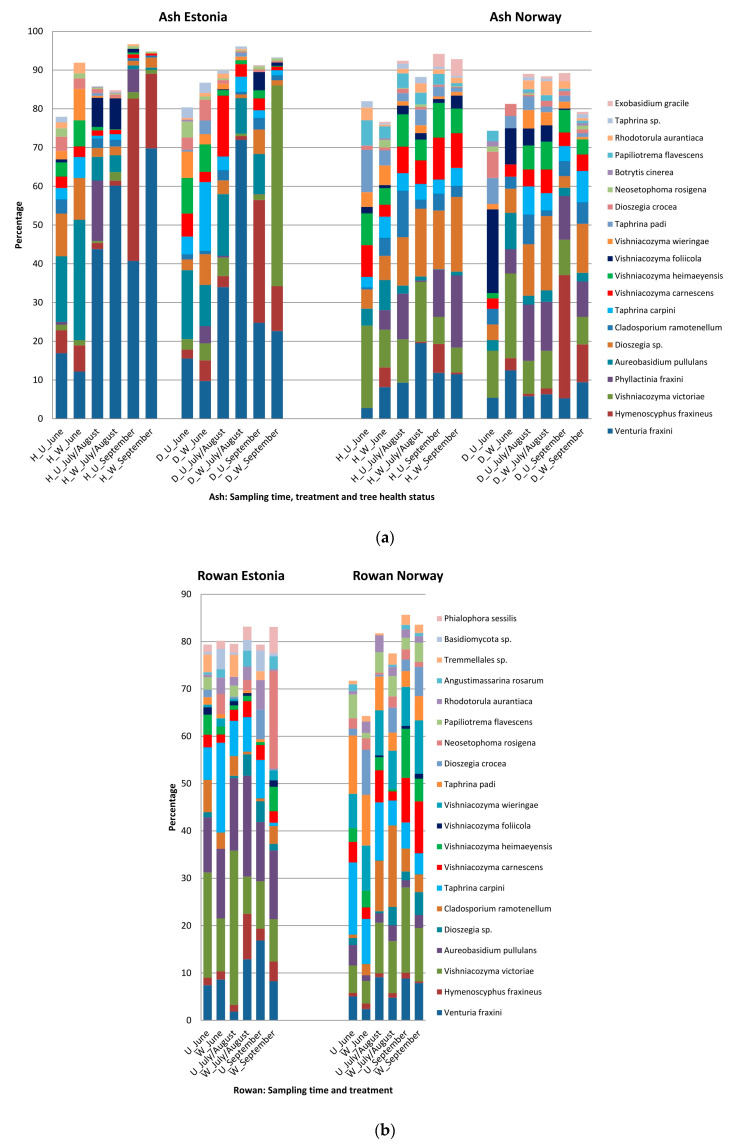
Across-season changes in relative proportions of the 20 most common species on ash (**a**) and rowan (**b**) from PacBio data H, healthy ash; D, diseased ash; U, unwashed; W, washed.

On ash, the 20 most common species included *Venturia fraxini* Aderh, *H. fraxineus*, nine species representing basidiomycetous yeasts in genera *Vishniacozyma*, *Dioeszegia* and *Rhodotorula*, five biotrophic species in genera *Phyllactinia*, *Taphrina* and *Exobasidium*, and four species in genera *Aureobasidium*, *Cladosporium*, *Botrytis*, *Neosetophoma* (Figure 3a; Supplementary Materials Tables S1 and S2). Together the read percentages of the 20 most common species covered 48% to 97% of all the sequence reads in Estonia and 76% to 94% in Norway, the lowest total read percentage of these species being in general in the samples collected on 30th June. When considering the average across-the-season read proportion of these 20 species for unwashed and washed leaflets, in the healthy trees the read proportions were higher in unwashed than washed leaflets, 88% and 73% for Estonia, and 90% and 87% for Norway, respectively. An opposite pattern was observed for the diseased trees, the average across-the-season read proportions of these 20 species from unwashed and washed leaflets being 86% and 88% for Estonia, and 83% and 85% for Norway, respectively (Figure 3). However, none of the abovementioned differences were statistically significant (*p* > 0.05).

The three most common fungi on ash in Estonia were *Venturia fraxini*, *H. fraxineus* and *Aureobasidium pullulans* de Bary., with average seasonal read proportion of 33%, 11% and 8%, respectively, and *Vishniacozyma victoriae* (M.J. Montes, Belloch, Galiana, M.D. Garca, C. Andrs, S. Ferrer, Torr.-Rodr. and J. Guinea), *Dioszegia* sp. and *V. fraxini* in Norway, with average seasonal read percentage of 11%, 10% and 9%, respectively. Fungi that showed significantly higher read percentage in Estonia than Norway included *V. fraxini* (*p* < 0.01), *H. fraxineus* (*p* < 0.05), *A. pullulans* (*p* < 0.01) and *Neosetophoma rosigena* Wanas. (*p* < 0.05). Fungi that showed significantly higher read percentage in Norway than Estonia included *Vishniacozyma victoriae* (*p* = 0.05), *Phyllactinia fraxini* de Candolle (Fuss). (*p* < 0.01), *Dioszegia* sp. (*p* < 0.01), *Cladosporium ramotenellum* K. Schub., Zalar, Crous and U. Braun, (*p* < 0.01), *Vishniacozyma heimayensis* (*p* < 0.01), *Papiliotrema flavescens* (Saito) (*p* < 0.01) and *Taphrina padi* (Jacz.) (*p* < 0.01). Excluding the difference in read percentage, the sites showed generally similar patterns for a given species. Concerning those species that showed an obvious seasonal pattern, the read percentages of *A. pullulans* peaked in June or July, those of *V. fraxini* and *P. fraxini* in late July or early August, and those of *H. fraxineus* in September. Concerning the washing treatment, the highest ratio between the read percentage from unwashed and washed ash leaflets was recorded for *H. fraxineus*, *C. ramotenellum*, *A. pullulans*, *V. carnescens*, *V. heimayensis* and *P. flavescens*, with respective values of 3.3, 1.9, 1.6, 1.4, 1.3 and 1.3 (Supplementary Materials Figure S5)—however, the higher read percentage from unwashed than washed leaflets was significant only for *V. heimayensis* (*p* = 0.03). The lowest ratio between the read percentage from unwashed and washed ash leaflets was recorded for *V. victoriae* and *V. fraxini*, with respective values of 0.5 and 0.7 (Supplementary Materials Figure S5)—the higher read percentage from washed than unwashed leaflets was significant only for *V. fraxini* (*p* = 0.05; Figure 3). Visualization of the ratio of read percent data between unwashed and washed ash leaflets by PCA clustered *H. fraxineus* apart from the other species (Supplementary Materials Figure S6). No species showed significantly higher read percentage on healthy ash trees than diseased ash trees, although for *P. flavescens*, a species detected only in Norway, the healthy trees showed generally higher read percentages than the diseased trees (*p* = 0.12). In contrast, the diseased ash trees had generally higher read percentage of *A. pullulans* than the healthy ash trees (*p* < 0.05). *Hymenoscyphus albidus*, the indigenous relative of *H. fraxineus*, was not found, although it was recognized based on the single test read that was added manually to the FASTA file in order to verify the resolution of our bioinformatics pipeline in distinguishing these two closely related species.

All the 20 most common fungal species on ash were detected on rowan as well, and 15 of those were also among the 20 most common species detected on rowan (Figure 3b; Supplementary Materials Tables S1 and S2). Species that were among the 20 most common ones on rowan but not among the 20 most common on ash were *Angustimassarina rosarum* Tibpromma, *Phialophora sessilis* de Hoog, *Xenoramularia neerlandica* Videira and Crous, an unidentified basidiomycete and an unidentified ascomycete, all with average sequence read percentages below 2%.

The three most common fungi on rowan were *V. victoriae*, *A. pullulans* and *V. fraxini* in Estonia, with average seasonal read percentage of 15%, 14% and 10%, respectively, and *V. victoriae*, *Vishniacozyma wieringae* and *Taphrina carpini* (Rostr.) in Norway, with average seasonal read percentage of 10%, 9% and 8%, respectively. The fungi that had generally higher read percentages in Estonia than in Norway on rowan were *V. fraxini* (*p* = 0.07), *H. fraxineus* (*p* < 0.01), *A. pullulans* (*p* < 0.01), *N. rosigena* (*p* = 0.07), *Angustimassarina rosarum* (*p* < 0.01), the first four were also more common on ash in Estonia than in Norway (Figure 3). Fungi that showed generally higher read percentage in Norway than in Estonia included *Dioszegia crocea* (Buhagiar) (*p* < 0.01), *V. wieringae* (*p* < 0.01), *P. flavescens* (*p* < 0.01), *C. ramotenellum* (*p* < 0.01), and *T. padi* (*p* < 0.01), the last three were also more common in ash in Norway than in Estonia. Seasonally, *P. fraxini* and *T. carpini* showed the highest read percentages in June, whereas *Phialophora sessilis*, a species detected only in Estonia, showed the highest read percentages in September (Figure 3b). Concerning the washing treatment, the highest ratios between read percentages from unwashed and washed rowan leaflets were recorded for *V. carnescens*, *V. victoriae*, an unidentified basidiomycete, *V. foliicola* and *V. heimayensis*, with respective values of 2.7, 1.8, 1.7, 1.6 and 1.5. However, the higher read percentage from unwashed than washed leaflets was significant only for *V. carnescens* (*p* < 0.05). The lowest ratio between read percentages from unwashed and washed rowan leaflets were recorded for *N. rosigena*, *P. sessilis*, *D. crocea* and *H. fraxineus*, with respective values of 0.3, 0.4, 0.4 and 0.4 (Supplementary Materials Figure S3)—the higher read percentage from washed than unwashed leaflets was significant only for *H. fraxineus* (*p* = 0.09). Visualization of the ratio of read percent data between unwashed and washed rowan leaflets by PCA did not cluster together any specific species (Supplementary Materials Figure S6). Considering the comparison of ash and rowan, the sequence read proportion of *H. fraxineus* on unwashed leaflets of ash (22.3% ± 2.62) (mean ± SE) exceeded that recorded on rowan by nearly 15-fold (1.5% ± 0.17%) (mean ± SE) (F_1.72_ = 6.0; R^2^_adj_ = 0.304; *p* = 0.037; Figure 3) in September. On washed leaflets, the proportion of *H. fraxineus* sequences was nearly 4.5 times higher on ash (15.5% ± 1.82%) (mean ± SE) than on rowan (3.5% ± 0.41%) (mean ± SE) in September (F_1.72_ = 11.0; R^2^_adj_ = 0.444; *p* = 0.0001). On other dates, no significant differences in *H. fraxineus* sequence proportion between tree species occurred in unwashed leaflets or leaflets subjected to washing (*p* > 0.05). The sequence read proportion of *V. fraxini* on unwashed leaflets of ash (17.8% ± 2.09%) (mean ± SE) exceeded that recorded on rowan on 30th June by nearly 4-fold (4.6% ± 0.54%) (mean ± SE) (F_1.72_ = 3.2; R^2^_adj_ = 0.124; *p* < 0.05; Figure 3). The proportion of *V. fraxini* showed no significant differences between ash and rowan in washed samples (*p* > 0.05) in any of the dates, however there was a trend in June towards ash (22.8% ± 2.68%) (mean ± SE) having higher percentage of *V. fraxini* than rowan (10.8% ± 1.27%) (mean ± SE) (*p* = 0.059; Figure 3).

The species co-occurrence analysis showed that the concomitant changes in read proportions of classifiable species across the samples were mostly random associations. Three species showed significant (*p* < 0.05) negative associations with *H. fraxineus* on ash leaflets from Estonia: *Taphrina padi* (*p* = 0.013), *Vishniacozyma laurentii* (*p* = 0.045) and *Rhodosporidiobolus colostri* (T. Castelli) (*p* < 0.001). No species detected on ash or rowan leaflets from Norway and on rowan leaflets from Estonia showed negative associations with *H. fraxineus*. The fungi with significant positive association with *H. fraxineus* (*p* < 0.05) on ash in Estonia included *V. victoriae*, *Bulleribasidium variabile* (Nakase and M. Suzuki) and unidentified species assigned to genera *Ramularia*, *Dioszegia* among others. No negative or positive associations were observed between *V. fraxini* and other species present.

### 3.5. Germination of H. fraxineus Ascospores on Detached Leaflets

In the experiment where *H. fraxineus* ascospores were discharged on ash leaflets in laboratory conditions, a large number of ascopores had germinated and penetrated leaf epidermis already 34 h after spore deposition. One or several germ tubes were formed per ascospore, the germ tubes infecting the leaflet directly or with the aid of appressorium-like structures. Infections were observed both on veinal and interveinal regions (Supplementary Materials Figure S8), no affinity towards stomata was observed. No exploratory hyphal growth along the leaf surface by germinated ascospores was observed even 96 h after the start of the experiment. Concerning other fungi present on the leaflets, hyphae with intercalary chlamydospores were commonly observed, primarily on veinal regions (Supplementary Materials Figure S9).

## 4. Discussion

### 4.1. Mycobiomes of Co-Inhabiting Ash and Rowan Are Shaped Similarly by Local Conditions

While several prior studies have considered the leaf mycobiome of European ash, to our knowledge this is the first overview of fungi present on the leaves of rowan, a common tree species in Europe and Asia. The leaflet mycobiomes of ash and rowan from the same site were highly similar, several prevalent fungi being more common at one site or the other, irrespective of the tree species. The majority of tree foliage associated fungi are considered to be non-specific [71], consistent with the observed high overlap in fungal species composition between ash and rowan. Based on fungal culturing from surface sterilized leaflets, Reiher [42] showed a high similarity in fungal species composition between co-inhabiting European ash, sycamore maple (*Acer pseudoplatanus* L.), common oak (*Quercus robur*) and small-leaved lime (*Tilia cordata* Mill.) at a forest stand in Germany free of ash dieback at the time of sampling. A high overlap in fungal species composition was recorded also between leaflets of co-inhabiting European ash and sycamore maple in a study from Switzerland based on next generation sequencing [44].

The high similarity in leaflet mycobiomes between ash and rowan within a site may reflect site-specific differences in the microclimate of the sampled tree canopies. The leaflets were sampled essentially at a comparable height from ash and rowan, 2–4 m above ground level at both sites. However, the trees sampled in Norway were understory trees in a forestland, receiving little direct sun light, while the trees sampled in Estonia grew in a more open spacing on former agricultural land, and the lower canopy layers were exposed to direct sunlight. Reiher [42] showed that fungal species richness associated with ash leaves is higher in understory trees than in canopy layers exposed to the sun. Some of the site-specific differences in species composition may be due to the fact that the 2nd sampling was done two weeks later in Norway (11th of August) than in Estonia (28th of July) whereas the third sampling was done two weeks earlier in Norway. This also means that the time period between 2nd and 3rd sampling was 1 month longer in Estonia. Therefore, PCoA analysis clustered samples from the 1st and 2nd sampling more closely together in Estonia, while samples from 2nd and 3rd sampling clustered more closely together in Norway (Figure S4).

### 4.2. Epiphytic Propagules Form a High Proportion of Leaflet Mycobiomes

The comparison of mycobiomes derived from unwashed and washed leaflets was pursued to consider the pool of fungal propagules occurring freely on leaflet surface and that attached firmly to leaf surface or residing within leaf tissues. The approach now used for washing, agitation of leaflets in water supplemented with a surfactant to dislodge epiphytic propagules, has been commonly used to study the propagule level of foliicolous yeasts on leaf surface, the procedure being coupled with plating a dilution series of the wash suspension onto appropriate media to determine colony forming units of yeasts [72]. Unwashed leaflets of ash showed a generally higher level of extrapolated total fungal DNA level than washed ash leaflets, indicating that a large proportion of ash leaf associated fungi reside on leaf surface. The extrapolation of total fungal biomass was achieved with the aid of qPCR based DNA amount estimate of *H. fraxineus* and the sequence read proportion of this fungus in a given sample. It should be noted that the approach assumes that the standard curve used for estimation of *H. fraxineus* DNA amount is directly applicable to all fungi, i.e., both the genome size and the copy number of *ITS* rDNA gene cluster are similar across fungal species. Since both traits can vary between species, caution should be exercised when interpreting a difference in extrapolated total fungal DNA amount estimates between samples of interest, and these kind of extrapolates should be regarded as rather directional than absolute. In this particular case, where we compared essentially highly complementary samples, i.e., unwashed leaflet vs. the corresponding washed leaflet of the same leaflet pair, we would expect the conclusion of unwashed leaflets hosting higher level of fungi than washed leaflets being robust.

While the read percentage of *H. fraxineus* was generally higher from unwashed than washed leaflets, the trend was not statistically significant. We also considered the epiphytic pool of *H. fraxineus* propagules with the aid of a specific qPCR assay. The unwashed leaflets of ash, collected on 11th August or 1st September in Norway, showed significantly higher qPCR estimates for *H. fraxineus* DNA amount than washed leaflets, whereas no such pattern was observed for ash leaflets sampled in Estonia. This site-specific difference but also the lower level of total fungal DNA and similar amount of *H. fraxineus* on healthy and diseased trees in Estonia than in Norway most likely reflect differences in leaflet sampling. In Estonia, a 1 cm^2^ piece of tissue was taken randomly from the sampled leaflet pairs and processed for DNA isolation as parallel samples for comparison of the treatment (i.e., washing vs. intact sample) effect, while in Norway the entire leaflets of sampled leaflet pairs were processed. Leaf colonizing microbes tend to be concentrated in aggregates, and leaf veins are frequently reported as hot spots of microbial colonization, this being attributed to increased water and nutrient availability in veinal regions [73]. Affinity to veinal regions would seem to apply to *H. fraxineus* as well, since in ash leaf tissues the highest levels of its DNA are associated with necrotic lesions on leaf veins [74]. Therefore, sampling of small tissue areas may involve more substantial sample-to-sample variation in the level of tissue colonization by both the indigenous fungi and *H. fraxineus* than processing of entire leaflets. The tendency for higher levels of *H. fraxineus* DNA in unwashed than washed leaflets from Norway, as determined by qPCR and DNA sequencing, would indicate that propagules residing on leaf surface form a significant proportion of the biomass of *H. fraxineus* associated with ash leaf tissues. The relatively high proportion of *H. fraxineus* sequence reads in washed rowan leaflets could be explained if part of its epiphytic propagules could not be removed by agitation in a water/surfactant solution. The chemical composition of the mucilage secreted by *H. fraxineus* ascospores on ash leaf surface [75] and its water solubility remain to be determined. The observations from the experiment where detached leaflets of ash were subjected to deposition of *H. fraxineus* ascospores showed that a number of these propagules can remain on the leaflet surface after heating at 90 °C in alkaline or acidic solutions. It remains to be examined to what extent the pathogen propagules, that can be removed by washing with a water/surfactant solution, represent ungerminated ascospores that have failed to establish infection or conidia, as the latter was observed on leaflet surface in growth chamber inoculation experiments mimicking natural infection by *H. fraxineus* ascospores [75].

For the indigenous fungi, we considered the pool of epiphytic propagules only by comparing their read percentages between unwashed and washed leaflets. Since sequence read percentage data is relative, it does not allow any conclusions about propagule levels in different samples. Ash leaflet associated total fungal biomass has been shown to increase many fold in August, and it seems obvious that several fungi, in addition to *H. fraxineus*, contribute to this increase [45]. Whether considering seasonal trajectory or a difference between treatments like unwashed vs. washed leaflets, sequence read data are conclusive only in the case the read percentage of a given species differs significantly between the compared samples. This was the case for the basidiomycetous yeast *Vishniacozyma heimayensis* on ash and its conspecific *V. carnescens* on rowan, which showed significantly higher sequence read percentages in unwashed than washed leaflets, the data indicating that epiphytic propagules form a significant pool of their biomass on the respective leaflets. Species with a nonsignificant trend for higher read percentages from unwashed than washed leaflets of ash or rowan included the dematiaceous hyphomycete *Cladosporium ramotenellum*, the basidiomycetous yeasts *Vishniacozyma victoriae* and *Vishniacozyma foliicola*, and the dimorphic fungus *Aureobasidium pullulans*. These data are in line with the notion that filamentous fungi are generally transient inhabitants of leaf surfaces, whereas rapidly sporulating hyphomycetes, dimorphic species, [76,77] and yeasts appear to colonize this habitat more actively [72].

### 4.3. Epiphytic Colonization—Niche Properties, Species Traits and Methodological Issues

The composition and the size of epiphytic microbial populations are shaped by the availability of nutrients, organic and inorganic molecules leaching from plant leaves, such as sugars, organic acids, amino acids, methanol and various salts [78,79] forming a major carbon source for epiphytic microbes. As reviewed by Fonseca and Inácio [72], the abundance of such nutrients varies with plant species, leaf age and growing conditions, the ability of foliicolous yeast species to grow on oligotrophic substrates being attributed to their exceptionally high affinity uptake systems for sugars and amino acids. Due to the limitations of sequence read proportion data, quantitative methods are required to determine the changes in population size of epiphytic species on ash leaflets. In this context it is also noteworthy, that the community structure of epiphytes depends on the carbon utilization profiles of species, the now detected *Vishniacozyma* species showing variation in their ability to assimilate specific carbon sources such as starch [80]. Both mechanical wounding and pathogen infection are known to facilitate the leaching of nutrients from leaves, the affected plant leaves supporting higher yeast populations compared with healthy leaves [81]. Compared to epiphytic yeasts, *Hymenoscyphus fraxineus* may be less dependent on nutrients present on the leaf surface, since the ascospores infect leaf tissues directly without any exploratory growth on the leaf surface. The rapidity of leaf infection by *H. fraxineus*, less than 48 h, as observed by Mansfield et al. [82] but also noted in the present study, suggests that the process is enabled by endogenous energy reserves present in the ascospore. To what extent leaf infection by *H. fraxineus* impacts the amount and composition of nutrients leaking from infected ash leaves remains to be examined. The generally considerably higher extrapolates for total fungal DNA amount in leaflets from diseased trees, compared to leaflets from healthy trees from Norway, would be consistent with fungal community responses to pathogen infection accelerated leaching of nutrients.

The property that makes leaf surface a harsh environment, is the frequent, repeated, rapid shifts in humidity, temperature and radiation. Traits specific to leaf surface inhabiting fungi, such as *A. pullulans* and foliicolous yeasts, include the production of an extracellular polysaccharide matrix that is thought to facilitate survival and growth in oligotrophic environments like the leaf surface. In yeasts, these capsules secure attachment, provide protection against desiccation, allow efficient rehydration following periods of drought, and also bind nutrients, thus contributing to higher growth rates of encapsulated yeast cells versus non-capsulated variants on nutrient-poor media [83]. Some areas in the natural habitat of *H. fraxineus*, in East Asia, have a continental climate with extremely long and hot drought periods in summer, so the fungus probably has also evolved defense mechanisms against desiccation and dehydration, which in turn, during seasons with dry summer, can give it an advantage over indigenous species inhabiting ash leaf surfaces in Europe. Coupled with the conspicuous melanization of ascospores after ejection [28], the mucilage secreted by *H. fraxineus* ascospores on ash leaf surface [75] could be one of the traits that contribute to survival of the pathogen propagules in this environment.

The predominance of basidiomycetous yeasts in unwashed leaflets of European ash was recorded also by next generation sequencing based mycobiome profiling by Bakys et al. [49], Cross et al. [45] and Griffiths et al. [46]. The fourth prior next generation sequencing based study of ash leaf mycobiome [44] involved surface-sterilization of leaflets with bleach (NAOCl) and 70% ethanol prior to DNA isolation, and recorded low sequence read proportions of basidiomycetous yeasts in ash leaflets. Ethanol is considered a broad solvent as its molecular structure allows for the dissolving of both hydrophilic and hydrophobic, and polar and nonpolar compounds [84], and the disparity between these studies could suggest that a surface sterilization procedure removes efficiently epiphytic yeast propagules. The fact that high sequence read percentages for yeasts were recorded also in washed leaflets in the present study could suggest that a water/surfactant solution is less efficient in removal of epiphytic propagules than ethanol.

### 4.4. Seasonal Trajectory of Species Richness and Impact of the Ash Dieback Pathogen

In June, the leaflets of rowan had significantly higher species richness than ash at both sites, which could relate to differences in the timing of flushing. Flushing of European ash takes place up to four weeks later than that of rowan in Northern Europe [85]. Fungal communities associated to leaves of deciduous plants generally undergo pronounced seasonal changes [7]. While some fungi associated with ash leaves can possibly overwinter in leaf buds [86], ash leaf tissues show a strong temporal recruitment pattern, the diversity of associated fungi increasing vigorously during early season [42].

The qPCR and PacBio sequence read data indicated that both the biomass of *H. fraxineus* and its relative proportion out of total fungal biomass associated with ash leaflets increased towards autumn, in synchrony with pathogen ascospore production (Figure 3; Figure S3). Along with the increase in *H. fraxineus* propagules on ash leaflets, the overall fungal richness declined significantly in September when compared to mid-season (July/August). In contrast, rowan showed a steady increase in fungal richness across the vegetation period. Studies carried out at a stand free of ash dieback, and based on fungal culturing from surface-sterilized tissues, showed a continuous increase in fungal species diversity in leaflets of European ash between May and October [42,43]. The now observed trajectory suggests that *H. fraxineus* disturbs the natural succession of ash leaf mycobiome in autumn, a period when endophytes with weak parasitic activity typically resume growth as a response to host tissue weakening by autumn senescence [71]. The low sporulation level of native ash leaf associated fungi during the peak sporulation period of *H. fraxineus* [45] is consistent with a model that these fungi spread early in the growing season and that their propagule level in planta remains below the carrying capacity of leaves until autumn senescence. The strong mid-season feedback from the saprobic phase to the parasitic period may be crucial for *H. fraxineus* to challenge the resident fungal community. One could envisage that the apparent disparity in ash leaf associated biomass between *H. fraxineus* and most of the indigenous fungi prior to the onset of leaf senescence, owing to the strong feedback from the saprotrophic phase of the invader, may provide *H. fraxineus* with an advantage when it comes to substrate capture and interference competition mediated by allelochemicals. In laboratory conditions, the antibiotic hymenosetin secreted by *H. fraxineus* showed broad spectrum antibacterial and antifungal activities [87].

The species co-occurrence analysis suggested that most of the interactions between *H. fraxineus* and indigenous fungi on ash and rowan leaflets were random. The read percentages of the basidiomycetous yeasts *Rhodosporidiobolus colostri* and *P. laurentii* and the dimorphic ascomycete *Taphrina padi*, known to cause pocket plum gall in *Prunus padus* [88], showed negative abundance associations with *H. fraxineus* on ash samples collected from Estonia, whereas in the Norwegian samples, the same species had no negative association with *H. fraxineus*. *Taphrina* species depend on living host tissues to complete the life cycle, and their sequence read percentages in community profiling studies have been observed to peak early in the season, both in ash [45] and oak leaves [89]. Similar trends were also seen in our study (Figure 3), and the negative association between *T. padi* and *H. fraxineus* probably reflects temporal niche differentiation. Both *R. colostri* and *P*. *laurentii* were seldom found in the study and therefore their potential role in supressing the development of *H. fraxineus* is questionable. Read percentages of twelve OTUs showed a significant positive association with *H. fraxineus* on ash at the Estonian site, these including *Ramularia* sp. and *Dioszegia* sp., among others. The evidence of positive associations involve species, which increase proportionally on ash along with the increase in *H. fraxineus*. Whether the observed co-increases result from changes in availability of resources owing to senescence and/or *H. fraxineus* infection related processes remains to be examined.

In the current work, *H. albidus*, a native relative of *H. fraxineus*, was not detected in either of the sampling sites, which would be consistent with the conclusion of prior studies that this species is being out-competed by *H. fraxineus* [41,44,90]. The life cycle of *H. fraxineus* is similar to *H. albidus*, which also is associated with ash leaf tissues but does not cause any shoot dieback [91]. The stroma of both species occupy the same spatial niche, inner cortex of leaf veins [92], but *H. fraxineus* grows faster than *H. albidus* in both leaf and woody material of European ash [93], which may facilitate competitive exclusion of *H. albidus*. There are no historical records of *H. albidus* in Estonia [51], but compared to *H. fraxineus*, *H. albidus* has very low occurrence frequency of ascomata [92], so it is not clear whether it has been outcompeted now but simply overlooked before the introduction of *H. fraxineus* to Estonia. The disappearance of *H. albidus* is consistent with this theory, according to which successful invaders should decrease the abundances mainly of species that are competitively similar to themselves [39]. A somewhat analogous scenario took place in relation to Dutch elm disease, where *Ophiostoma ulmi*, the causative agent of the disease during its first invasive phase, has been competitively eliminated by the second wave caused by *Ophiostoma novo-ulmi* [94].

### 4.5. Ash Phenotype and Fungal Diversity

We found that overall fungal species richness was statistically similar between symptomatic and asymptomatic European ash at both sampling sites. Other studies [44,45] did not find any differences in fungal species richness or level of *H. fraxineus* DNA associated with leaf tissues between ash genotypes with and without shoot dieback symptoms. In contrast, Griffiths et al. [46] reported that diversity, species composition and network structure of ash leaf microbial communities were positively associated with the severity of the ash dieback disease in a study that involved both saplings and mature trees, and proposed that high fungal species diversity may facilitate leaf infection by *H. fraxineus.* However, prior studies indicate that the lowest fungal diversity in ash leaflets is present in the light canopy of mature ash trees [42]. The level of *H. fraxineus* ascospores also decline drastically along with distance to the ground [95], a feature that was recorded also in the present study, and yet, also in large mature trees the top shoots are highly susceptible to the disease [96]. Could it be that there is a positive relation between leaf colonization level by indigenous species and the *H. fraxineus* spore load required to establish itself in the tissue? A systematic comparison of the fungal community size and diversity and *H. fraxineus* spore deposition level in leaflets collected from understory plants and light canopy of mature ash trees would be needed to clarify this.

In the present study *A. pullulans* showed a significantly higher read percentage on leaflets of ash trees with shoot dieback than on leaflets of healthy ash (Figure 3). While the specific localization of *A. pullulans* on ash leaflets remains to be established, in other plant species large epiphytic colonies of this fungus are found in veinal regions, whereas single cells are detected in interveinal areas, the difference being attributed to variation in resource availability [77]. Since *H. fraxineus* also has specific affinity towards leaf veins, this raises the question of the nature of the interaction between the invader and other fungi competing for this niche. *Aureobasidium pullulans* is found on different hosts, e.g., *Smilax rotundifolia* L. [97] and has shown antagonistic activity towards a number of phytopathogenic fungi [98], while in our study there was no negative (or positive) association between read percentages of *H. fraxineus* and *A. pullulans* on ash leaflets. In the study of Schlegel et al. [99], exudates from *A. pullulans* did not have any impact on *H. fraxineus* ascospore germination. In comparison to *Ophiostoma novo-ulmi*, the causative agent of the second wave of Dutch elm disease, and a group of elm endophytes, *A. pullulans* showed low efficiency in using various carbon sources, except for phenolics [100]. This would be consistent with trade-offs between adaptation to tolerate extreme environmental stress and utilization efficiency of easily accessible carbon sources. The full range of carbon sources that *H. fraxineus* is able to use has not been examined. Considering the low genomic variation present in the European population of the fungus, there is remarkable variation in the expression of cellulases, peroxidases, polyphenoloxidases and amylases between individual strains of *H. fraxineus* [101], something that may suggest complementary carbon utilization profiles among strains when it comes to assimilation of cellulose, phenolics and polysaccharides. This observation would be consistent with the finding that the two parental strains that founded the European population of *H. fraxineus* were obviously very divergent [102,103]. One possibility for the prevalence of *A. pullulans* in leaflets of diseased ash could be enhanced leakage of compounds for which the fungus has high utilization efficiency, such as phenolics, but the lack of any positive correlation between read percentages of *A. pullulans* and *H. fraxineus* does not provide any support to such a scenario. One alternative explanation could be phenotypic differences in leaf chemicals. While the constitutive phenolics present in ash leaflets did not discriminate between trees that show phenotypic differences in susceptibility to shoot infection [104], the leaves of ash trees with high susceptibility to ash dieback were, in comparison to leaves from tolerant ash trees, enriched in iridioid glycosides [105]. *Aureobasidium pullulans* is able to hydrolyse glycosidic terpenes [106], but whether there is any positive relation between enhanced production of antifeeding terpenoids and *A. pullulans* remains to be examined.

### 4.6. Niche Partitioning and Host Specificity of Hymenoscyphus fraxineus and Venturia fraxini

The high prevalence of the indigenous necrotroph *V. fraxini* in ash leaflets already in June, obviously as endophytic thalli as no symptoms were observed in ash leaflets at this time or in late July/early August, deserves specific consideration. The knowledge about *V. fraxini* in comparison to *H. fraxineus* is very limited, probably because *V. fraxini* seems to be a harmless leaf pathogen. While *V. fraxini* is also reported to be associated primarily with leaf veins, its thin stroma are formed beneath the cuticle or within the epidermis [107]. In contrast, the ascomata of *H. fraxineus* arise from a stroma formed in the inner cortex of leaf veins, next to the supportive sclerenchyma tissue [92]. This would suggest that *V. fraxini* and *H. fraxineus* occupy different spatial niches, which is consistent with the persistence of *V. fraxini* even at stands with a long history of ash dieback.

In comparison to rowan, the number of indigenous fungi with high specificity to ash appears low. *Hymenoscyphus fraxineus* and *Venturia fraxini*, considered to be specific to ash, were common on rowan as well. The same sequence variant of *H. fraxineus* was present on ash and rowan. Concerning *V. fraxini*, there were in total three different sequence variants present in our dataset, the most common, *V. fraxini* 1 (99.7% of all *V. fraxini* sequences), was prevalent in both ash and rowan while the less common variants, *V. fraxini* 2 (0.2% of all *V. fraxini* sequences) and *V. fraxini* 3 (0.1% of all *V. fraxini* sequences), were only found in ash. Recently, no *H. fraxineus* sequences were detected on sycamore maple (*Acer pseudoplatanus*) included as a reference tree in an ash mycobiome study in Switzerland, and the *Venturia* sequences detected on this tree were different from those of *V. fraxini* [44]. This could simply be a consequence of the ethanol surface sterilization used by the authors in case it efficiently removed all surface propagules of *H. fraxineus* and *V. fraxini*. However, leaf characteristics such as structure, shape, surface hairs and exudates play a significant role in spore deposition [108]. It is possible that the shape and the presence of hairs on the abaxial surface of rowan leaflets, resembling those of European ash leaflets, render rowan leaflets more favourable to *H. fraxineus* and *V. fraxini* spore interception than the large and hairless leaf blades of sycamore maple. In consistency, European ash and the studied rowan (*S. aucuparia*) are more efficient in atmospheric particle entrapping than sycamore maple [109].

An alternative explanation for the high occurrence of *H. fraxineus* and *V. fraxini* on rowan is that these species are less host-specific than previously considered. Some fungi are known to occur as leaf endophytes on a broad range of hosts but sporulate only on one or a few of them [110]. *Fusarium circinatum*, an invasive ascomycete causing pine pitch canker, is able to cause endophytic infections on grass and herb species growing under diseased *Pinus radiata* trees at stands with high pathogen infection pressure [111]. Could it be that *V. fraxini* and *H. fraxineus* can survive on certain additional tree species but sporulate only on ash? Reiher [42] isolated *V. fraxini* also from surface-sterilized leaves of common oak (*Quercus robur*) growing together with European ash, whereas Junker [112] showed that *H. fraxineus* is capable of causing necrosis on the herb basil (*Ocimum basilicum* L., Lamiaceae family of order Lamiales) in laboratory trials. Further, in the UK *H. fraxineus* has been detected from symptomatic leaf and shoot tissues of three ornamentals in the Oleaceae family of order Lamiales, the evergreen narrow-leaved mock privet (*Phillyrea angustifolia* L.) and mock privet (*Phillyrea latifolia* L.), and the deciduous white fringetree (*Chionanthus virginicus* L.) (https://www.forestresearch.gov.uk/news/chalara-ash-dieback-different-ash-species-and-non-ash-hosts/, Ana Perez-Sierra, Forest Research, UK, pers. comm. 25.05.2020). The relation between evolution of polyextremotolerant species like *A. pullulans* and opportunistic infections of these species on novel hosts such as humans was recently discussed by Gostinčar et al. [113]. In analogy, unless any infection established by *H. fraxineus* on a non-ash species was able to produce conidia or ascospores, such opportunistic infections may be considered meaningless from an evolutionary (but also from an epidemiological) perspective. However, if the pathogen was able to return to ash from the additional hosts, any newly acquired adaptations would be either beneficial, neutral or detrimental for its association with ash. The range of additional hosts and their significance in the life cycle of *H. fraxineus* (and *V. fraxini*) remain to be clarified.

### 4.7. Methodological Aspects

In this work, we show that third generation sequencing (PacBio) works well for community studies, and for precise species detection as also emphasized by Castaño et al. [114]. Because PacBio yields longer sequence reads, this in turn allows more precise species identification, as showed also in other recent studies [55,56]. According to Tedersoo et al. [57], PacBio data was 16% more efficient (on average) in identification of the genus level compared to Illumina MiSeq, but this advantage decreased when comparing results at class and phylum level. They ascribed this advantage to either a lower level of sequencing artefacts that are known to accumulate with sequencing depth [115], or to the scenario that previous Sanger sequencing based studies failed to capture rare species [116]. Interestingly, some differences were detected between qPCR and PacBio in relation to *H. fraxineus*. While PacBio detected *H. fraxineus* in ash leaflets already in samples collected on 30th June, qPCR did not, this suggesting that PacBio has a lower detection limit. A similar observation was made by Cross et al. [45], who compared Ion Torrent sequencing of *ITS2* and qPCR for detection of *H. fraxineus*. An *ITS1catta* primer [54] was designed originally for this study in order to circumvent the relatively long intron in the end of *Hymenoscyphus 18S rRNA* gene. This long intron is known to cause problems in retrieving *H. fraxineus* sequences with standard *ITS1* targeting primers. For example, *Hymenoscyphus* species were not captured by the primer sets used by Cross et al. [45] for amplification of the *ITS1* region. Griffiths et al. [46] were also not able to detect *H. fraxineus* in their study using Illumina sequencing platform. Further emphasizing the bias associated with standard *ITS1* or *ITS2* targeting primers, Cross et al. [45] did not detect *V. fraxini* even though the experimental stand was the same as now examined. Though we did not find any *H. albidus* reads or other *Hymenoscyphus* species (apart from *H. fraxineus*) in our study, the manually added *H. albidus* sequence showed that the *ITS* region worked well in differentiating these two sister species. Although the number of reads is lower in the case of PacBio sequencing and there may be some length bias towards longer reads being better represented, our study shows that more precise species detection ability and longer read lengths render this platform to a suitable tool in studies of fungal phytopathogens.

Besides the biases associated with the choice of primers and sequencing platform, also the decision whether to wash, surface sterilize, or process leaf tissues directly to DNA extraction may significantly affect the resolution of a study to capture the range of foliicolous fungi and the associated spatial partitioning of the niche. To further the understanding of the community dynamics and species interrelations, next generation sequencing coupled with qPCR and carbon source utilization profiling of species of interest seems warranted.

## 5. Conclusions

The main findings of this study showed that mycobiomes of ash and rowan from the same site were highly similar but showed a different seasonal trajectory. The overall fungal richness on European ash leaves declined significantly along with an estimated increase in *H. fraxineus* biomass. On rowan, as might be expected for an undisturbed community, the fungal richness steadily increased across the vegetation period.

Indigenous fungi implicated to have a large proportion of their propagules on leaflet surface included *Vishniacozyma victoriae*, *Vishniacozyma foliicola* and *Aureobasidium pullulans*, yeasts adapted to survive on harsh oligotrophic substrates and capitalizing on nutrients leaching from plant tissues. For some of the detected filamentous fungi like the biotroph *Phyllactinia fraxini* and the hyphomycete *Cladosporium ramotenellum*, a large proportion of their propagules represent presumably asexual spores supported by mycelia feeding within leaf tissues. The tendency for higher qPCR estimates and read percentages for *H. fraxineus* from unwashed than washed ash leaflets indicated that a significant proportion of the propagules of *H. fraxineus* also reside on the surface of leaflets in late summer. It remains unclear to what extent the pathogen propagules residing on leaf surface represent dormant ascospores—ascospores that have failed to germinate or asexual spores supported by thalli feeding within leaf tissues. Deciphering the nature and potential roles of the epiphytic propagules of *H. fraxineus* in pathogenesis and interaction with indigenous fungi is warranted.

We also did not find any significant differences in *H. fraxineus* DNA level between leaflets of healthy and symptomatic ash trees, suggesting that differences in the degree of shoot dieback among phenotypes are not due to any differential level of leaf infection by the pathogen.

It should be noted that the interaction between indigenous fungi and *H. fraxineus* in phyllosphere represents only the first stage of their relationship—it is becoming increasingly obvious that leaf endophytes are part of the decomposer community as well. No data is currently available about the successional stages in ash leaf litter. A study conducted in Ukraine at a site free of ash dieback at the time of sample collection showed that *A. pullulans* and *V. fraxini* were the most prevalent fungi in overwintered ash petioles [50]. Considering the extensive *H. fraxineus* pseudosclerotia that often cover the entire rachis/petiole tissue [92], one might expect that the obviously strong saprobic competence of *H. fraxineus* impacts fungal community dynamics also during leaf decomposition [117]. Ascomata of *H. fraxineus* can be formed not only in the year after leaf fall but also in older petioles, at least up to five growing seasons after the leaves have been shed [118]. This feature, another extremophilic trait of this fungus, testifies for a strong competence in defending the saprobic niche against abiotic and biotic stress. The long persistence of *H. fraxineus* in shed petioles suggests that the fungus may indeed interfere also with the succession of saprobic fungal community. Thus, to fully understand the interactions between *H. fraxineus* and the native fungal community, profiling of the microbial succession also during leaf litter decomposition is warranted.

## Supplementary Materials

The following are available online at https://www.mdpi.com/2309-608X/6/3/150/s1, Figure S1: Sampling design: sampling site, tree species and sampling date—the number of samples subjected to sequencing is indicated within parenthesis, Figure S2: Collection of compound leaves of ash and rowan, and sampling of leaflets for DNA extraction. At both sites, compound leaves were collected across the season from selected and marked trees within each of the following groups: (1) two ash trees showing obvious signs of *H. fraxineus* infection in their shoots, (2) two ash trees without any shoot symptoms, and (3) two rowan trees. In both countries, one compound leaf per tree and time point was collected for DNA extraction two randomly chosen leaflets pairs per compound leaf were used: in Estonia, a 1 cm^2^ piece was taken from the sampled leaflets next to the mid-rib vein, while in Norway the entire leaflets were processed. For each of the sampled leaflet pairs, one sample was subjected to washing prior to DNA extraction and the other was processed without washing. The rectangulars on the ash leaflets show an example of how two leaflet pairs were sampled in Estonia (In Norway the corresponding entire leaflets would have been sampled), Figure S3: Amount of *Hymenoscyphus fraxineus* DNA (ng DNA/mg leaflet tissue) on ash and rowan in Estonia (a) and Norway (b) and ascospore levels of *H. fraxineus* in the Norwegian stand through the vegetation period of 2014—data show the level of airborne pathogen ascospores from midnight to noon per day, two sampling heights (ground level and 5 m above ground) were used (c). Leaf material collected in Norway: level of *H. fraxineus* DNA in unwashed (uw) and washed (w) leaflets of healthy ash (H), ash with shoot dieback symptoms (D) and rowan (R) used for qPCR screening to choose dates for sequencing. (concerning samples from the three dates subjected to sequencing (30.06., 11.08., 01.09.), *n* =4 per tree, phenotype and treatment for ash and *n* = 3 per treatment for rowan, for the other dates, *n* = 2 per per tree, phenotype and treatment. For dates with missing value, no pathogen DNA was detected. In Estonia unwashed samples of ash and rowan were used to choose the dates for sequencing (*n* = 4 per tree, per date), Figure S4: PCoA plots for fungal species composition between tree species and different sampling dates on the Norwegian (a and b) and Estonian site (c and d) on both ash and rowan samples, Figure S5: The sequence read for each fungal taxon between unwashed and washed samples of *Fraxinus excelsior* (*H. fraxineus* is marked as X and *V. fraxini* as a circle; (a) and the read for each fungal taxon between unwashed and washed samples of *Sorbus aucuparia* (*Vishniacozyma victoriae* is marked as a triangle; (b), Figure S6: PCA plot of read ratio between unwashed and washed leaflets for 20 most common species on ash (a) and rowan (b), Estonia and Norway data merged, Figure S7: Across-season changes in relative proportions of fungal classes across the dataset on ash (a) and rowan (b) in Estonia, Figure S8: Across-season changes in relative proportions of fungal classes across the dataset on ash (a) and rowan (b) in Norway, Figure S9: *H. fraxineus* ascospores on detached leaflet of *F. excelsior*. Photo taken 34 h after ascospore deposition, note the germ tubes and appressoria-like swellings, Figure S10: Hyphae of indigenous species growing on veinal regions of ash leaflets and showing intercalary chlamydospores, Table S1: Abundance, growth form in vegetative phase and hypothetical functional group of fungal species detected on leaflets of *Fraxinus excelsior* and *Sorbus aucuparia*. For growth form, F indicates filamentous, Y, yeast and D dimorphic. For the hypothetical functional group, En indicates endophytic, Ep epiphytic, B biotrophic, M mycoparasitic, N necrotrophic and S saprotrophic, Table S2: Relative abundance of fungal species present on unwashed and washed leaflets of *Fraxinus excelsior* (a) and *Sorbus aucuparia* (b).
