# Supplementary material for: The Relationship between Fungal Diversity and Invasibility of a Foliar Niche—The Case of Ash Dieback
Source: J Fungi (Basel). 2020 Aug 26;6(3):150. doi: 10.3390/jof6030150 (PMC7558441; doi:10.3390/jof6030150)
Supplement: Supplementary file 1 [file jof-06-00150-s001.pdf]

Supplementary material

Table S1. Abundance, growth form in vegetative phase and hypothetical functional group of fungal species detected on leaflets of *Fraxinus excelsior* and *Sorbus aucuparia.* For growth form, F indicates filamentous, Y, yeast and D dimorphic. For the hypothetical functional group, En indicates endophytic, Ep epiphytic, B biotrophic, M mycoparasitic, N necrotrophic and S saprotrophic

Growth form	Hypothetical Functional group	Species	UNITE species hypotheses	No of sequences on ash/rowan	% of ash/rowan sequences
F	N	*Venturia fraxini*	SH131889.07FU	2136/196	23.70/8.0
F	N	*Hymenoscyphus fraxineus*	SH106453.07FU	936/52	10.39/2.12
Y	Ep/En	*Vishniacozyma victoriae*	SH085564.07FU	743/335	8.25/13.67
Y	Ep/En	*Dioszegia sp*		736/52	8.17/2.12
F	B	*Phyllactinia fraxini*	SH111028.07FU	613/9	6.80/0.37
D	Ep/En	*Aureobasidium pullullans*	SH103321.07FU	465/204	5.16/8.32
Y	Ep/En	*Vishniacozyma heimaeyensis*	SH085568.07FU	385/97	4.27/3.96
Y	Ep/En	*Vishniacozyma carnescens*	SH1159265.08FU	382/44	4.24/1.8
F		*Cladosporium ramotenellum*	SH129886.07FU	314/162	3.48/6.61
D	B	*Taphrina carpini*	SH100611.07FU	302/119	3.35/4.86
Y	Ep/En	*Vishniacozyma foliicola*	SH085570.07FU	263/22	2.92/0.9
Y	Ep/En	*Filobasidium wieringae*	SH134145.07FU	134/132	1.49/5.39
D	B	*Taphrina padi*	SH1153419.08FU	129/82	1.43/3.35
Y	Ep/En	*Dioszegia aurantica*	SH116917.07FU	92/46	1.02/1.88
Y	Ep/En	*Papilioderma flavescens*	SH113230.07FU	89/48	0.99/1.96
D	B	*Taphrina sp.*		54/18	0.60/0.73
F	N	*Botrytis cinerea*	SH1189120.08FU	51/104	0.57/4.24
F	B	*Exobasidium gracile*	SH116261.07FU	40/3	0.44/0.12
Y	Ep/En	*Sporobolomyces ruberrimus*	SH102276.07FU	39/5	0.43/0.20
F		*Septoria sp.*		35/10	0.39/0.41
D		*Tilletiopsis sp.*		30/10	0.33/0.41
Y	Ep/En	*Hannaella coprosmae*	SH1155517.08FU	30/3	0.33/0.12
F	M	*Angustimassarina rosarum*	SH1182782.08FU	29/39	0.32/1.59
Y	Ep/En	*Vishniacozyma dimennae*	SH112907.07FU	28/7	0.31/0.29
Y	Ep/En	*Dioszegia fristingensis*	SH120007.07FU	25/8	0.28/0.33
F	N	*Didymella sp.*		24/8	0.27/0.33
?	B	*Microstroma juglandis*	SH092670.07FU	24/1	0.27/0.04
F	En	*Dothiora pyrenophora*	SH1149665.08FU	23/21	0.26/0.86
F	S	*Russula vitellina*	SH090222.07FU	22/0	0.24/0
F		*Leptosphaeria rubefaciens*	SH124988.07FU	19/2	0.21/0.08

Growth form	Hypothetical Functional group	Species	UNITE species hypotheses	No of sequences on ash/rowan	% of ash/rowan sequences
Y	Ep/En	*Dioszegia sp.*		18/0	0.20/0
		Ascomycota sp.		17/1	0.19/0.04
		Helotiales sp.		17/0	0.19/0
F		*Phaeosphaeria sp.*		16/13	0.18/0.53
F	N	*Alternaria infectoria*	SH128560.07FU	16/5	0.18/0.20
F	N	*Ramularia rumicicola*	SH1194043.08FU	16/3	0.18/0.12
F		*Xenoramularia neerlandica*	SH1194047.08FU	15/1	0.17/0.04
Y	Ep/En	*Dioszegia butyracea*	SH120009.07FU	15/3	0.17/0.12
F		*Naevala minutissima*	SH089113.07FU	14/4	0.16/0.16
F	N	*Ramularia cynarae*	SH1194043.08FU	13/10	0.14/0.41
		Cadophora sp.		13/4	0.14/0.16
F	N	*Fusarium tricinctum*	SH131965.07FU	12/9	0.13/0.37
F		*Sclerostagonospora sp.*	SH1157054.08FU	12/4	0.13/0.16
		Ascomycota sp.		11/4	0.12/0.16
Y	Ep/En	*Vihniacozyma globospora*	SH112906.07FU	10/13	0.11/0.53
Y		*Knufia sp*		10/2	0.11/0.08
F	S	*Neosetophoma rosigena*	SH1157057.08FU	9/9	0.10/0.37
Y	Ep/En	*Vishniacozyma foliicola*	SH1159266.08FU	9/0	0.10/0
F	B	*Pucciniastrum areolatum*	SH1227098.08FU	9/7	0.10/0.29
Y	EP/En	*Itersonilia pannonica*	SH107249.07FU	9/1	0.10/0.04
Y	Ep/En	*Sporobolomyces roseus*	SH1192588.08FU	9/0	0.10/0
F		*Ramularia hydrangeae-macrophyllae*	SH116484.07FU	8/6	0.09/0.24
F	B	*Exobasidium sp*		8/4	0.09/0.16
Y		*Cryptococcus flavescens*	SH114390.07FU	8/4	0.09/0.16
Y	Ep/En	*Bulleribasidium variabile*	SH135101.07FU	8/3	0.09/0.12
		Basidiomycota sp.		7/31	0.08/1.26
F	N	*Davidiella tassiana*	SH1190878.08FU	7/4	0.08/0.16
Y	Ep/En	*Bullera alba*	SH126915.07FU	7/4	0.08/0.16
F		*Dothiora sp.*		7/1	0.08/0.04
F	N	*Venturia fraxini*	SH131889.07FU	6/0	0.07/0
F		*Pleosporales sp.*		6/0	0.07/0
		Ascomycota sp.		6/0	0.07/0
F		*Cladosporium sp.*		5/0	0.06/0
F		*Xenoramularia neerlandica*	SH1194047.08FU	5/16	0.06/0.65
F		*Loratospora luzulae*	SH1157055.08FU	5/2	0.06/0.08
F	B	*Melampsora sp.*		5/0	0.06/0
F	S	*Trametes versicolor*	SH100234.07FU	5/0	0.06/0
		Ascomycete sp		4/20	0.04/0.82
F		*Alternaria sp.*		4/5	0.04/0.20
Y	Ep/En	*Rhodosporidiobolus colostri*	SH1181847.08FU	4/7	0.04/0.29

Growth form	Hypothetical Functional group	Species	UNITE species hypotheses	No of sequences on ash/rowan	% of ash/rowan sequences
		Ascomycota sp.		4/3	0.04/0.12
Y		*Cryptococcus sp.*		4/5	0.04/0.20
D	B	*Taphrina sadebeckii*	SH100610.07FU	4/1	0.04/0.04
F	B	*Thekopsora areolata*	SH1227098.08FU	4/3	0.04/0.12
F		*Pleosporales sp*		4/1	0.04/0.04
F	N	*Monographella nivalis var nivalis*	SH124583.07FU	4/0	0.04/0
D	Ep/En	*Aureobasidium pullulans*	SH103321.07FU	4/0	0.04/0
F	B	*Podosphaera tridactyla*	SH081935.07FU	4/0	0.04/0
F		*Pleosporales sp*		3/10	0.03/0.41
D		*Ophiostoma quercus*	SH135043.07FU	3/2	0.03/0.08
F	N	*Colletotrichum acutatum*	SH117167.07FU	3/3	0.03/0.12
Y	Ep/En	*Rhodotorula pinicola*	SH1214242.08FU	3/5	0.03/0.20
F	N	*Sphaerulina berberidis*	SH1194042.08FU	3/3	0.03/0.12
F	N	*Microcyclosporella mali*	SH1201747.08FU	3/0	0.03/0
Y		*Knufia cryptophialidica*	SH101062.07FU	3/1	0.03/0.04
Y		*Filobasidium sp.*		3/0	0.03/0
F		*Cladosporium sp.*		3/3	0.03/0.12
		Ascomycota sp.		2/3	0.02/0.12
F		*Exophiala sp.*		2/4	0.02/0.16
Y	Ep/En	*Pseudomicrostroma phylloplanum*	SH1185854.08FU	2/3	0.02/0.12
Y	Ep/En	*Bulleribasidium variabile*	SH135101.07FU	2/3	0.02/0.12
F		*Tetracladium sp.*		2/1	0.02/0.04
F		*Phaeosphaeria sp.*		2/4	0.02/0.16
F	S	*Peniophora cinerea*	SH1235335.08FU	2/2	0.02/0.08
F		*Sarcinomyces sp.*		2/2	0.02/0.08
F	N	*Parastagonospora nodorum*	SH1157054.08FU	2/2	0.02/0.08
Y		*Malassezia globosa*	SH088577.07FU	2/1	0.02/0.04
D	N	*Ophiostoma novo ulmi*	SH135046.07FU	2/0	0.02/0
F	N	*Gibellulopsis nigrescens*	SH1234027.08FU	2/1	0.02/0.04
F	N	*Venturia fraxini*	SH131889.07FU	2/0	0.02/0
D	Ep/En	*Dioszegia takashimae*	SH086148.07FU	2/0	0.02/0
D	Ep/En	*Aureobasidium pullullans*	SH1149661.08FU	2/0	0.02/0
F	S	*Gloeophyllum trabeum*	SH105175.07FU	2/0	0.02/0
Y		*Cystobasidium sp.*		2/0	0.02/0
F		*Alternaria metachromatic*	SH1142798.08FU	2/0	0.02/0
F	B	*Blumeria graminis*	SH114813.07FU	2/0	0.02/0
F		*Massarina sp*		2/0	0.02/0
F	N	*Phialophora sessilis*	SH1228270.08FU	1/32	0.01/1.31
Y		*Malassezia restricta*	SH1149886.08FU	1/2	0.01/0.08

Growth form	Hypothetical Functional group	Species	UNITE species hypotheses	No of sequences on ash/rowan	% of ash/rowan sequences
F		*Muriformistrickeria rubi*	SH1234973.08FU	1/5	0.01/0.20
Y		*Filobasidium sp.*		1/4	0.01/0.16
F	En	*Lophodermium piceae*	SH129503.07FU	1/4	0.01/0.16
		Ascomycota sp.	SH1222417.08FU	1/3	0.01/0.12
F		*Penicillium bialowiezense*	SH107620.07FU	1/2	0.01/0.08
Y		*Dioszegia rishiriensis*	SH120016.07FU	1/2	0.01/0.08
		Helotiales sp.		1/1	0.01/0.04
F		*Fusarium sp.*		1/0	0.01/0
		Ascomycete sp		1/0	0.01/0
Y		P*hyllozyma coprosmicola*	SH085547.07FU	1/1	0.01/0.04

Table S2. Relative abundance of fungal species present on unwashed and washed leaflets of *Fraxinus excelsior* (a) and *Sorbus aucuparia* (b)

a

Species	Unwashed	Washed	% of unwashed sequences	% of washed sequences
Venturia fraxini	1067	1069	20.34	28.41
Hymenoscyphus fraxineus	769	167	14.66	4.44
Phyllactinia fraxini	387	226	7.38	6.01
Dioszegia sp.	370	366	7.05	9.73
Aureobasidium pullulans	321	144	6.12	3.83
Cryptococcus victoriae	295	448	5.62	11.91
Cryptococcus carnescens	286	152	5.45	4.04
Cryptococcus heimaeyensis	237	131	4.52	3.48
Cladosporium ramotenellum	219	83	4.18	2.21
Taphrina carpini	162	152	3.09	4.04
Vishniacozyma foliicola	158	105	3.01	2.79
Taphrina padi	82	47	1.56	1.25
Cryptococcus wieringae	78	56	1.49	1.49
Cryptococcus flavescens	58	31	1.11	0.82
Dioszegia crocea	53	32	1.01	0.85
Rhodotorula aurantiaca	52	40	0.99	1.06
Exobasidium gracile	41	36	0.78	0.96
Taphrina sp.	32	22	0.61	0.58
Neosetophoma rosigena	31	20	0.59	0.53
Tremmellales sp.	24	13	0.46	0.35
Symmetrospora coprosmae	23	17	0.44	0.45
Sporobolomyces ruberrimus	23	16	0.44	0.43

Species	Unwashed	Washed	% of unwashed sequences	% of washed sequences
Angustimassarina rosarum	21	8	0.40	0.21
Bullera coprosmae	20	10	0.38	0.27
Sporobolomyces gracilis	18	19	0.34	0.50
Tilletiopsis sp	16	14	0.31	0.37
Cryptococcus heimaeyensis	15	2	0.29	0.05
Septoria sp.	14	21	0.27	0.56
Dioszegia fristingensis	14	11	0.27	0.29
Microstroma juglandis	14	10	0.27	0.27
Dothiora pyrenophora	13	10	0.25	0.27
Alternaria infectoria	12	4	0.23	0.11
Xenoramularia neerlandica	11	4	0.21	0.11
Didymella sp.	11	13	0.21	0.35
Ramularia rumicicola	11	5	0.21	0.13
Cryptococcus dimennae	10	18	0.19	0.48
Cadophora sp.	10	3	0.19	0.08
Sclerostagonospora sp.	10	2	0.19	0.05
Phaeosphaeria sp.	9	7	0.17	0.19
Ramularia cynarae	9	4	0.17	0.11
Bensingtonia yuccicola	9	5	0.17	0.13
Helotiales sp.	9	8	0.17	0.21
Dioszegia butyracea	8	7	0.15	0.19
Ascomycota sp.2	7	10	0.13	0.27
Vishniacozyma foliicola2	7	2	0.13	0.05
Bulleribasidium variabile	7	1	0.13	0.03
Bacidina mendax	7	1	0.13	0.03
Russula vitellina	6	16	0.11	0.43
Pucciniastrum areolatum	6	3	0.11	0.08
Sporobolomyces roseus	6	3	0.11	0.08
Pleosporales sp.	6	0	0.11	0.00
Ascomycota sp.5	6	0	0.11	0.00
Botrytis cinerea	5	4	0.10	0.11
Naevala minutissima	5	9	0.10	0.24
Bullera globospora	5	5	0.10	0.13
Davidiella tassiana	5	2	0.10	0.05
Knufia sp.	5	5	0.10	0.13
Exobasidium sp.	5	3	0.10	0.08
Papilioterma flavecens	5	3	0.10	0.08
Trametes versicolor	5	0	0.10	0.00
Basidiomycota sp.	4	3	0.08	0.08
Dioszegia sp.2	4	14	0.08	0.37
Alternaria sp.	4	0	0.08	0.00

Species	Unwashed	Washed	% of unwashed sequences	% of washed sequences
Bulleromyces albus	4	3	0.08	0.08
Venturia fraxini2	4	2	0.08	0.05
Monographella nivalis var nivalis	4	0	0.08	0.00
Peniophora cinerea	4	0	0.08	0.00
Ascomycota sp.	3	8	0.06	0.21
Ramularia hydrangeae-macrophyllae	3	5	0.06	0.13
Rhodosporidiobolus colostri	3	1	0.06	0.03
Itersonilia pannonica	3	6	0.06	0.16
Ascomycota sp.4	3	1	0.06	0.03
Colletotrichum acutatum	3	0	0.06	0.00
Xenoramularia neerlandica2	3	2	0.06	0.05
Cryptococcus sp.	3	1	0.06	0.03
Taphrina sadebeckii	3	1	0.06	0.03
Thekopsora areolata	3	1	0.06	0.03
Phoma sp.	3	0	0.06	0.00
Melampsora sp.	3	2	0.06	0.05
Podosphaera tridactyla	3	1	0.06	0.03
Ascomycete sp	2	2	0.04	0.05
Fusarium tricinctum	2	10	0.04	0.27
Ophiostoma quercus	2	1	0.04	0.03
Exophiala sp.	2	0	0.04	0.00
Loratospora luzulae	2	3	0.04	0.08
Pseudomicrostroma phylloplanum	2	0	0.04	0.00
Tetracladium sp.	2	0	0.04	0.00
Aureobasidium pullulans2	2	0	0.04	0.00
Knufia cryptophialidica	2	1	0.04	0.03
Parastagonospora nodorum	2	0	0.04	0.00
Ophiostoma novo ulmi	2	0	0.04	0.00
Filobasidium sp.2	2	1	0.04	0.03
Cladosporium sp.	2	1	0.04	0.03
Aerobasidium pullullans3	2	0	0.04	0.00
Blumeria graminis	2	0	0.04	0.00
Cystobasidium sp.	2	0	0.04	0.00
Phialophora sessilis	1	0	0.02	0.00
Pleosporales sp	1	2	0.02	0.05
Malassezia restricta	1	0	0.02	0.00
Ascomycota sp.3	1	1	0.02	0.03
Rhodotorula pinicola	1	2	0.02	0.05
Sphaerulina berberidis	1	2	0.02	0.05

Species	Unwashed	Washed	% of unwashed sequences	% of washed sequences
Bulleribasidium variabile	1	1	0.02	0.03
Microcyclosporella mali	1	2	0.02	0.05
Phaeosphaeria sp.2	1	1	0.02	0.03
Pleosporales sp2	1	3	0.02	0.08
Lophodermium piceae	1	0	0.02	0.00
Ascomycota sp.6	1	0	0.02	0.00
Malassezia globosa	1	1	0.02	0.03
Venturia fraxini3	1	1	0.02	0.03
Gibellulopsis nigrescens	1	0	0.02	0.00
Ascomycete sp2	1	0	0.02	0.00
Leptosphaeria rubefaciens	0	19	0.00	0.50
Cladosporium sp..	0	5	0.00	0.13
Muriformistrickeria rubi	0	1	0.00	0.03
Heterocephalacria sp.	0	2	0.00	0.05
Dothiora sp.	0	7	0.00	0.19
Filobasidium sp.	0	1	0.00	0.03
Penicillium bialowiezense	0	2	0.00	0.05
Dioszegia takashimae	0	2	0.00	0.05
Dioszegia rishiriensis	0	1	0.00	0.03
Gloeophyllum trabeum	0	2	0.00	0.05
Graphilbum sp.	0	1	0.00	0.03
Phyllozyma coprosmicola	0	2	0.00	0.05
Massarina sp	0	1	0.00	0.03
Helotiales sp.2	0	1	0.00	0.03
Alternaria metachromatic	0	2	0.00	0.05
Metschnikowia sp.	0	1	0.00	0.03
Cryptococcus heimaeyensis2	0	2	0.00	0.05

b

Species	Unwashed	Washed	% of unwashed sequences	% of washed sequences
Vishniacozyma victoriae	222	113	17.33	9.67
Venturia fraxini	104	92	8.12	7.87
Vishniacozyma carnescens	96	53	7.49	4.53
Taphrina carpini	93	69	7.26	5.90
Aureobasidium pullulans	91	113	7.10	9.67
Vishniacozyma wieringae	66	66	5.15	5.65
Vishniacozyma heimaeyensis	60	37	4.68	3.17
Cladosporium ramotenellum	54	65	4.22	5.56
Taphrina padi	48	34	3.75	2.91
Papilioderma flavescens	26	22	2.03	1.88
Rhodotorula aurantiaca	26	20	2.03	1.71
Neosetophoma rosigena	25	79	1.95	6.76
Angustimassarina rosarum	20	19	1.56	1.63
Basidiomycota sp.	20	11	1.56	0.94
Dioszegia sp.	18	34	1.41	2.91
Hymenoscyphus fraxineus	17	35	1.33	2.99
Dioszegia crocea	15	38	1.17	3.25
Vishniacozyma foliicola	14	8	1.09	0.68
Dothiora pyrenophora	14	7	1.09	0.60
Xenoramularia neerlandica	11	5	0.86	0.43
Tremmellales sp.	10	11	0.78	0.94
Phialophora sessilis	9	23	0.70	1.97
Dioszegia sp.2	9	7	0.70	0.60
Septoria sp.	8	2	0.62	0.17
Ascomycete sp	8	12	0.62	1.03
Ramularia cynarae	7	3	0.55	0.26
Bullera globospora	7	6	0.55	0.51
Pseudocercosporella sp.	7	10	0.55	0.86
Exobasidium arescens	7	5	0.55	0.43
Taphrina sp.	6	12	0.47	1.03
Didymella sp.	6	2	0.47	0.17
Phaeosphaeria sp.	6	7	0.47	0.60
Pleosporales sp	6	4	0.47	0.34
Phyllactinia fraxini	5	4	0.39	0.34
Botrytis cinerea	5	4	0.39	0.34
Vishniacozyma dimennae	5	2	0.39	0.17
Sporobolomyces gracilis	4	2	0.31	0.17
Exobasidium sp.	4	0	0.31	0.00
Rhodosporidiobolus colostri	4	3	0.31	0.26

Species	Unwashed	Washed	% of unwashed sequences	% of washed sequences
Podosphaera leucotricha	4	0	0.31	0.00
Dioszegia fristingensis	3	5	0.23	0.43
Alternaria infectoria	3	2	0.23	0.17
Dioszegia butyracea	3	0	0.23	0.00
Cadophora sp.	3	1	0.23	0.09
Fusarium tricinctum	3	6	0.23	0.51
Davidiella tassiana	3	1	0.23	0.09
Pucciniastrum areolatum	3	4	0.23	0.34
Alternaria sp.	3	2	0.23	0.17
Muriformistrickeria rubi	3	2	0.23	0.17
Heterocephalacria sp.	3	4	0.23	0.34
Vishniacozyma sp.	3	2	0.23	0.17
Exophiala sp.	3	1	0.23	0.09
Filobasidium sp.	3	1	0.23	0.09
Ascomycota sp.6	3	0	0.23	0.00
Microcyclospora tardicrescens	3	0	0.23	0.00
Symmetrospora coprosmae	2	1	0.16	0.09
Sporobolomyces ruberrimus	2	3	0.16	0.26
Bullera coprosmae	2	1	0.16	0.09
Naevala minutissima	2	2	0.16	0.17
Bensingtonia yuccicola	2	1	0.16	0.09
Ramularia hydrangeae-macrophyllae	2	4	0.16	0.34
Knufia sp.	2	0	0.16	0.00
Papilioterma flavecens	2	2	0.16	0.17
Ophiostoma quercus	2	0	0.16	0.00
Colletotrichum acutatum	2	1	0.16	0.09
Rhodotorula pinicola	2	3	0.16	0.26
Pseudomicrostroma phylloplanum	2	1	0.16	0.09
Sphaerulina berberidis	2	1	0.16	0.09
Taphrina wiesneri	2	1	0.16	0.09
Phoma sp.2	2	2	0.16	0.17
Parastagonospora nodorum	2	0	0.16	0.00
Mrakiella sp.	2	1	0.16	0.09
Fusarium sp.	2	0	0.16	0.00
Dioszegia zsoltii	2	0	0.16	0.00
Tilletiopsis sp	1	9	0.08	0.77
Ramularia rumicicola	1	2	0.08	0.17
Vishniacozyma heimaeyensis	1	0	0.08	0.00
Leptosphaeria rubefaciens	1	1	0.08	0.09

Species	Unwashed	Washed	% of unwashed sequences	% of washed sequences
Sclerostagonospora sp.	1	3	0.08	0.26
Cladosporium sp.	1	2	0.08	0.17
Malassezia restricta	1	1	0.08	0.09
Itersonilia pannonica	1	0	0.08	0.00
Ascomycota sp.3	1	2	0.08	0.17
Bulleromyces albus	1	3	0.08	0.26
Ascomycota sp.4	1	2	0.08	0.17
Bulleribasidium variabile	1	0	0.08	0.00
Loratospora luzulae	1	1	0.08	0.09
Dothiora sp.	1	0	0.08	0.00
Tetracladium sp.	1	0	0.08	0.00
Phaeosphaeria sp.2	1	3	0.08	0.26
Lophodermium piceae	1	3	0.08	0.26
Sarcinomyces sp.	1	1	0.08	0.09
Peniophora sp.	1	1	0.08	0.09
Malassezia globosa	1	0	0.08	0.00
Filobasidium sp.3	1	1	0.08	0.09
Curvibasidium pallidicorallinum	1	2	0.08	0.17
Boeremia exigua var. pseudolilacis	1	2	0.08	0.17
Penicillium bialowiezense	1	0	0.08	0.00
Dioszegia rishiriensis	1	1	0.08	0.09
Bannozyma sp.	1	1	0.08	0.09
Phylliscum sp.	1	1	0.08	0.09
Massarina sp	1	0	0.08	0.00
Helotiales sp.2	1	0	0.08	0.00
Physcia adscendens	1	1	0.08	0.09
Parmelia sulcata	1	0	0.08	0.00
Exobasidium gracile	0	1	0.00	0.09
Ascomycota sp.	0	4	0.00	0.34
Microstroma juglandis	0	1	0.00	0.09
Ascomycota sp.2	0	1	0.00	0.09
Xenoramularia neerlandica2	0	1	0.00	0.09
Taphrina sadebeckii	0	1	0.00	0.09
Thekopsora areolata	0	3	0.00	0.26
Phoma sp.	0	1	0.00	0.09
Bulleribasidium variabile	0	3	0.00	0.26
Pleosporales sp2	0	1	0.00	0.09
Aureobasidium pullulans2	0	2	0.00	0.17
Knufia cryptophialidica	0	1	0.00	0.09

Species	Unwashed	Washed	% of unwashed sequences	% of washed sequences
Gibellulopsis nigrescens	0	2	0.00	0.17
Rhizosphaera sp.	0	3	0.00	0.26
Symmetrospora sp.	0	1	0.00	0.09
Itersonilia perplexans	0	1	0.00	0.09
Graphilbum sp.	0	1	0.00	0.09
Resinicium bicolor	0	2	0.00	0.17
Genolevuria tibetensis	0	2	0.00	0.17

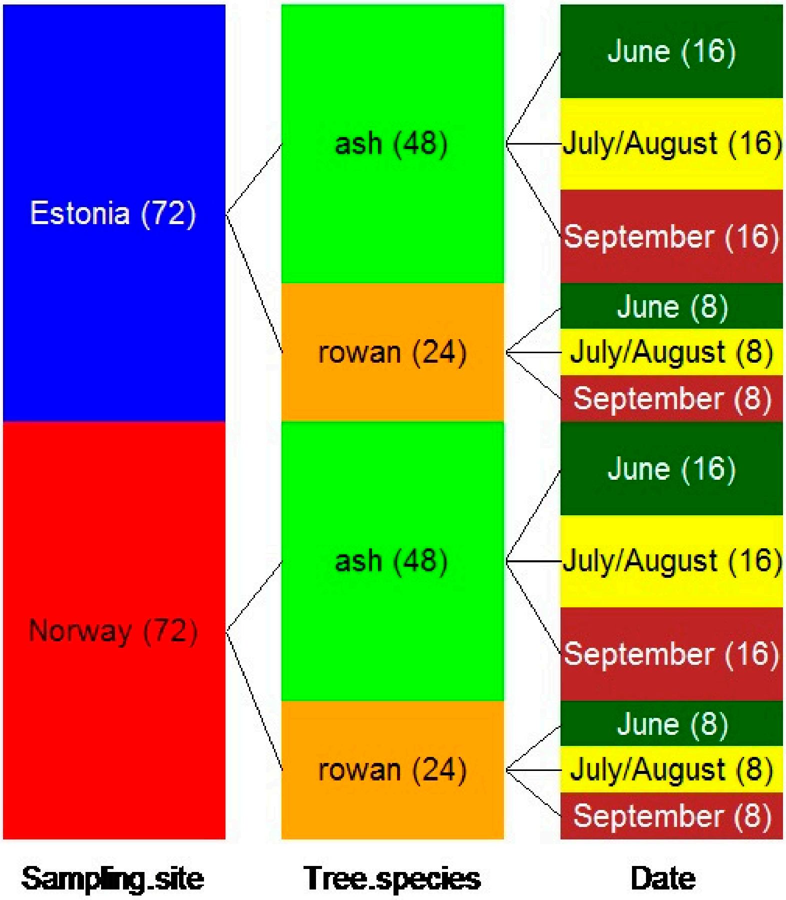

Figure S1. Sampling design: sampling site, tree species and sampling date – the number of samples subjected to sequencing is indicated within parenthesis

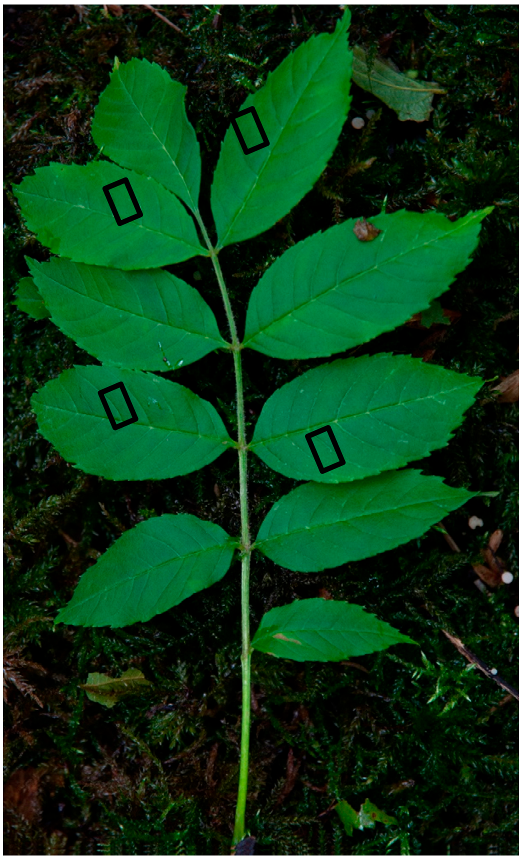

Figure S2. Collection of compound leaves of ash and rowan, and sampling of leaflets for DNA extraction. At both sites, compound leaves were collected across the season from selected and marked trees within each of the following groups: 1) two ash trees showing obvious signs of *H. fraxineus* infection in their shoots, 2) two ash trees without any shoot symptoms, and 3) two rowan trees. In both countries, one compound leaf per tree and time point was collected - for DNA extraction two randomly chosen leaflets pairs per compound leaf were used: in Estonia, a 1-cm2 piece was taken from the sampled leaflets next to the mid-rib vein, while in Norway the entire leaflets were processed. For each of the sampled leaflet pairs, one sample was subjected to washing prior to DNA extraction and the other was processed without washing. The rectangulars on the ash leaflets show an example of how two leaflet pairs were sampled in Estonia (In Norway the corresponding entire leaflets would have been sampled)

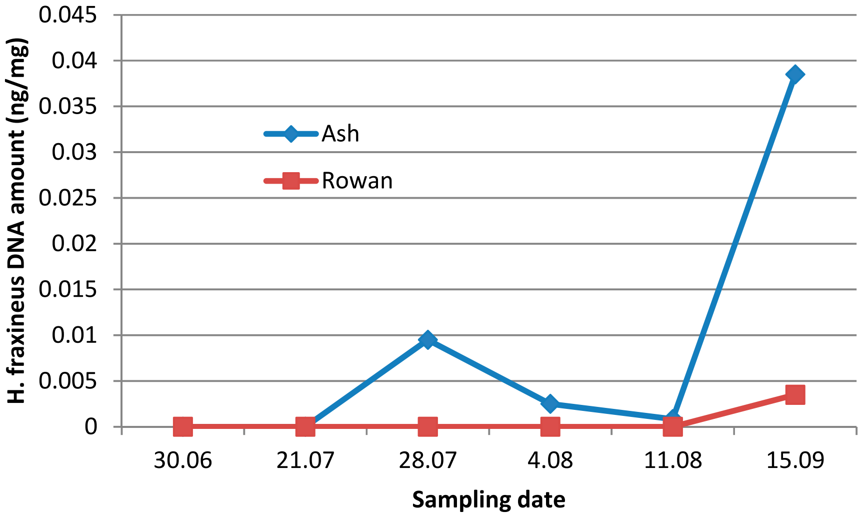
H. fraxineus DNA amount (ng/mg)
0.045
0.04
0.035
0.03
0.025
0.02
0.015
0.01
0.005
0
Ash
Rowan
30.06
21.07
28.07
4.08
11.08
15.09
Sampling date

a

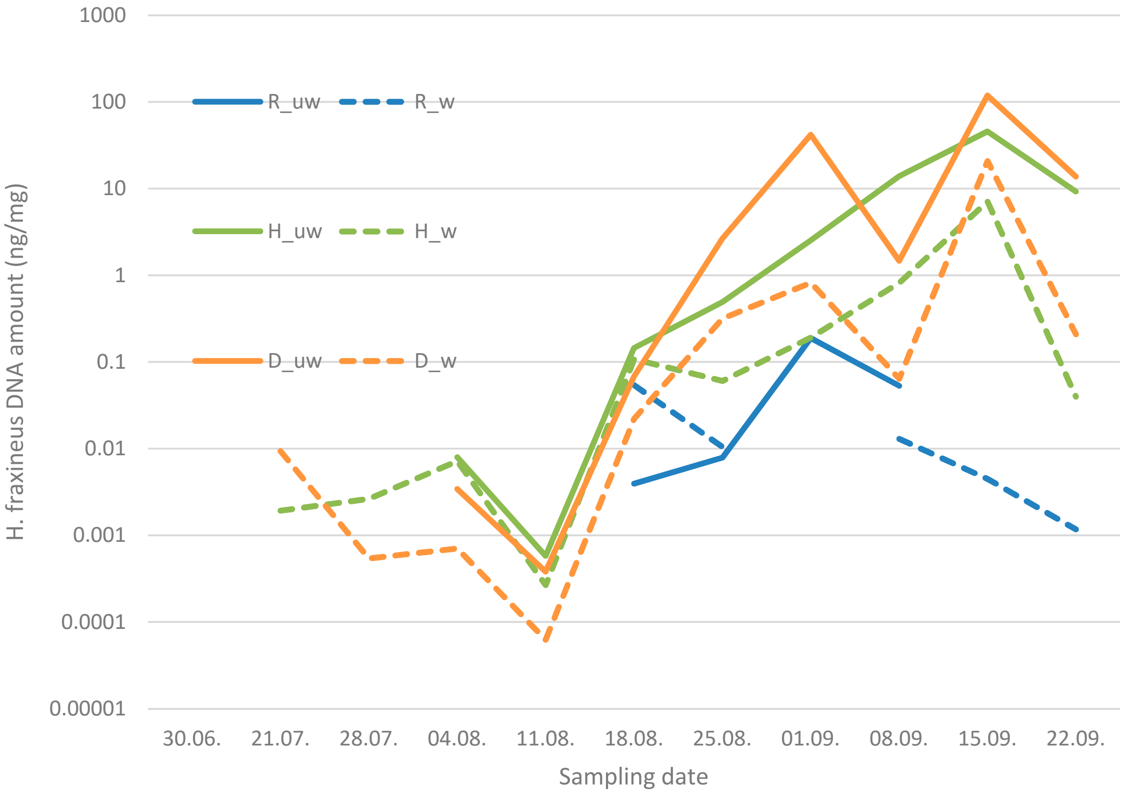
H. fraxineus DNA amount (ng/mg)
1000
100
10
1
0.1
0.01
0.001
0.0001
0.00001
R_uw
R_w
H_uw
H_w
D_uw
D_w
30.06.
21.07.
28.07.
04.08.
11.08.
18.08.
25.08.
01.09.
08.09.
15.09.
22.09.
Sampling date

b

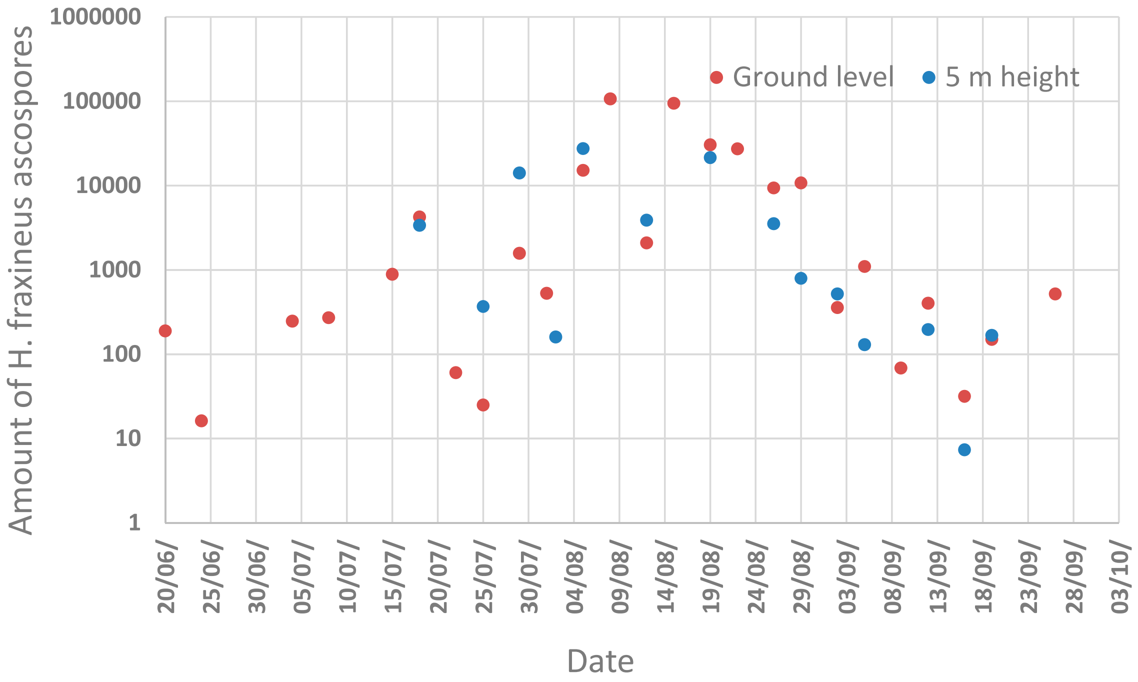

c

Figure S3. Amount of *Hymenoscyphus fraxineus* DNA (ng DNA/mg leaflet tissue) on ash and rowan in Estonia (a) and Norway (b) and ascospore levels of *H. fraxineus* in the Norwegian stand through the vegetation period of 2014 – data show the level of airborne pathogen ascospores from midnight to noon per day, two sampling heights (ground level and 5m above ground) were used (c). Leaf material collected in Norway: level of *H. fraxineus* DNA in unwashed (uw) and washed (w) leaflets of healthy ash (H), ash with shoot dieback symptoms (D) and rowan (R) used for qPCR screening to choose dates for sequencing. (concerning samples from the three dates subjected to sequencing (30.06., 11.08., 01.09.), n =4 per tree, phenotype and treatment for ash and n=3 per treatment for rowan, for the other dates, n=2 per per tree, phenotype and treatment. For dates with missing value, no pathogen DNA was detected. In Estonia unwashed samples of ash and rowan were used to choose the dates for sequencing (n=4 per tree, per date)

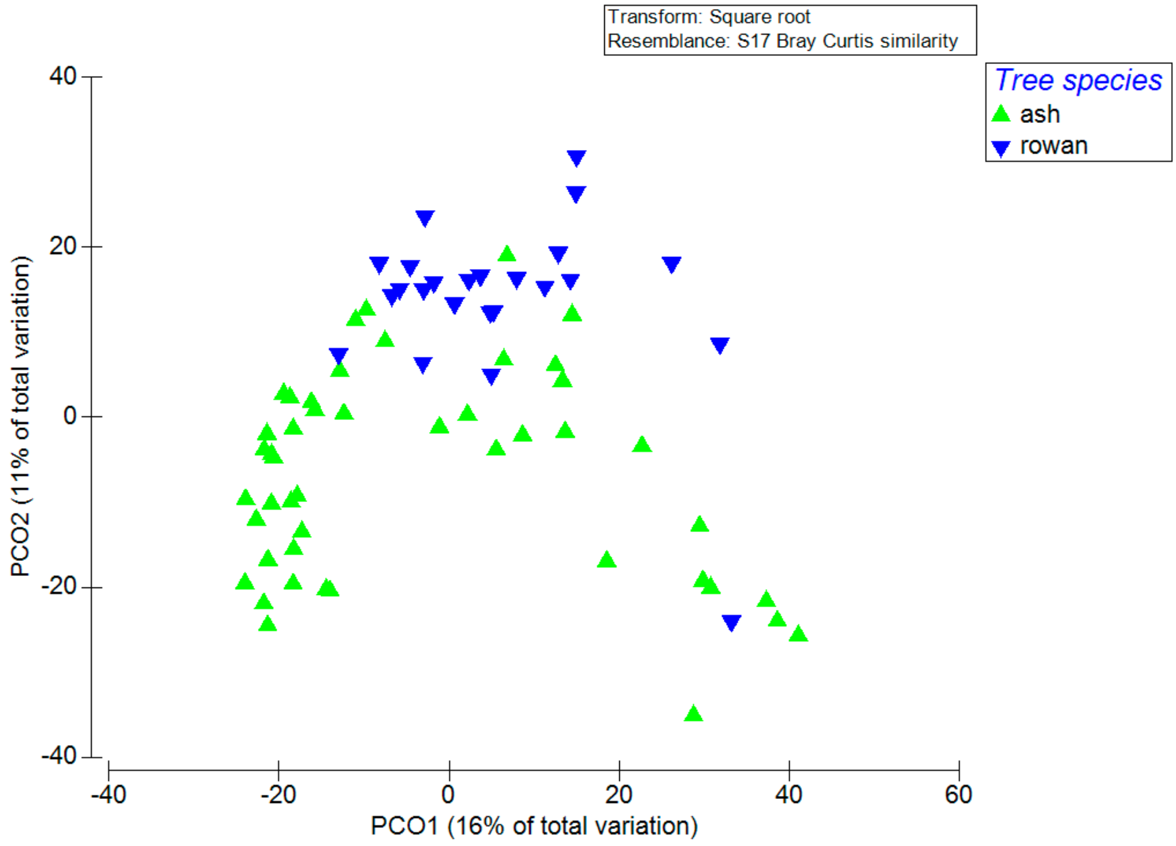
Transform: Square root
Resemblance: S17 Bray Curtis similarity
Tree species
ash
rowan
40
20
0
-20
-40
PCO2 (11% of total variation)
-40
-20
0
20
40
60
PCO1 (16% of total variation)

a

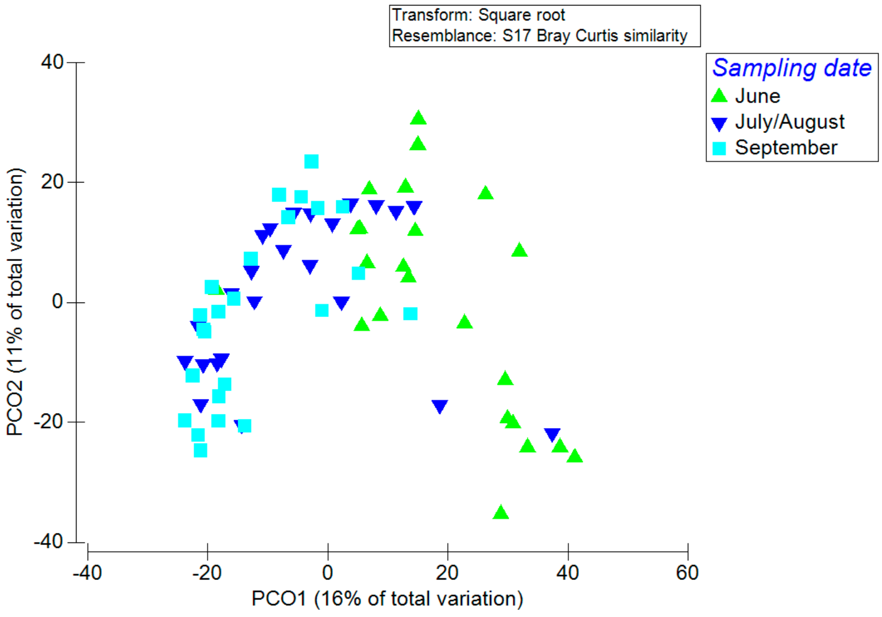
Transform: Square root
Resemblance: S17 Bray Curtis similarity
Sampling date
June
July/August
September
40
20
0
-20
-40
PCO2 (11% of total variation)
-40
-20
0
20
40
60
PCO1 (16% of total variation)

b

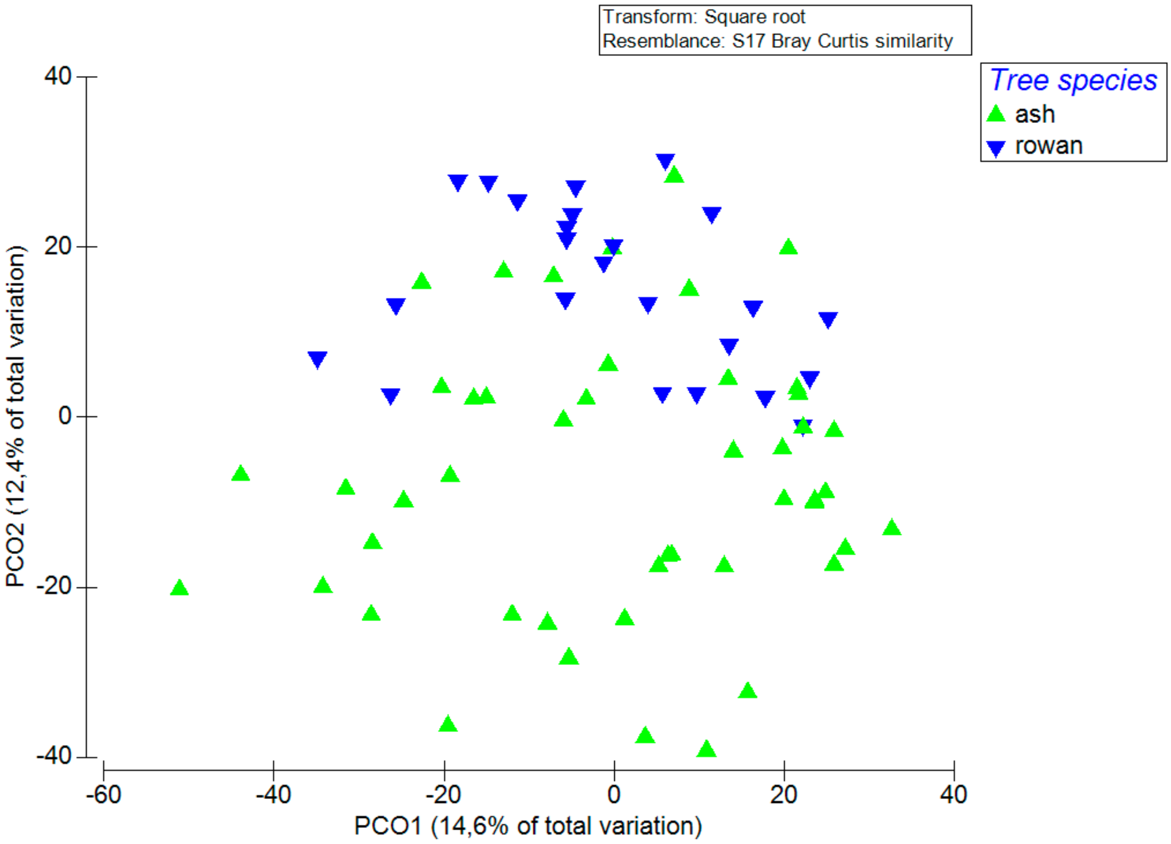

Transform: Square root
Resemblance: S17 Bray Curtis similarity
Tree species
ash
rowan
40
20
0
-20
-40
PCO2 (12,4% of total variation)
-60
-40
-20
0
20
40
PCO1 (14,6% of total variation)

c

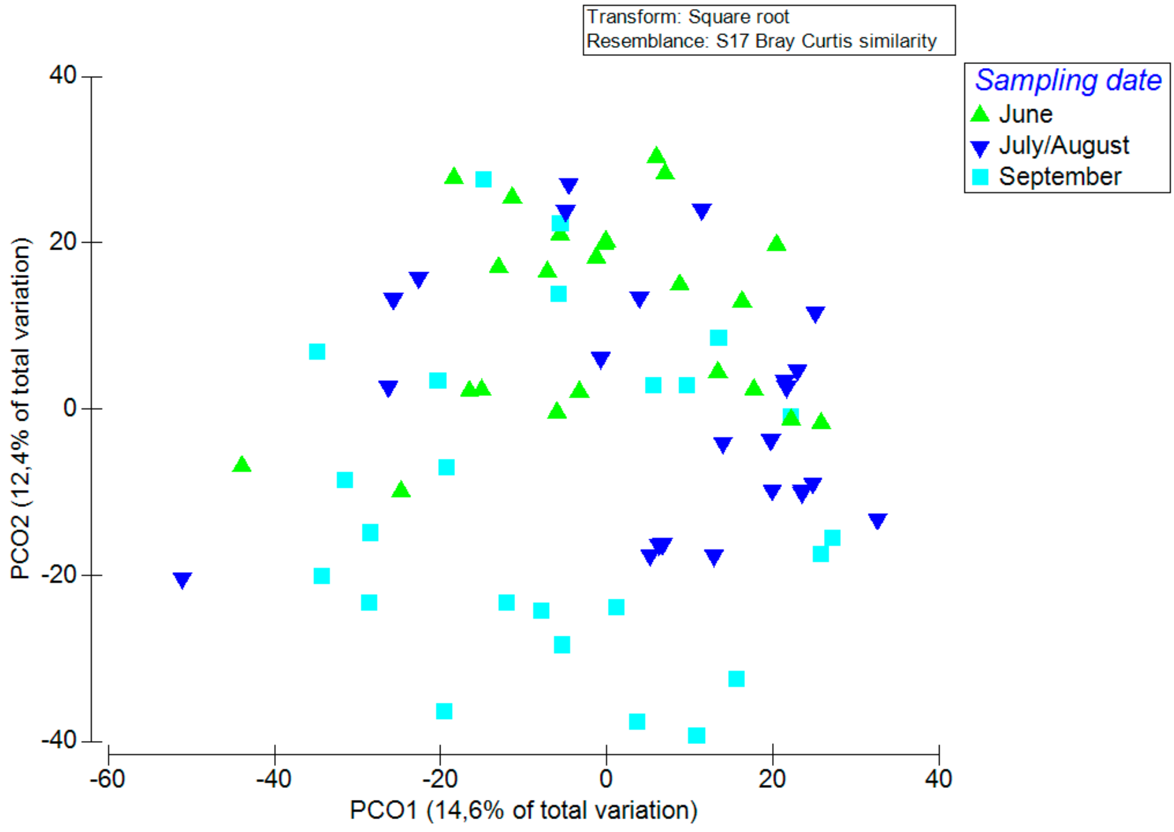

d

Figure S4. PCoA plots for fungal species composition between tree species and different sampling dates on the Norwegian (a and b) and Estonian site (c and d) on both ash and rowan samples

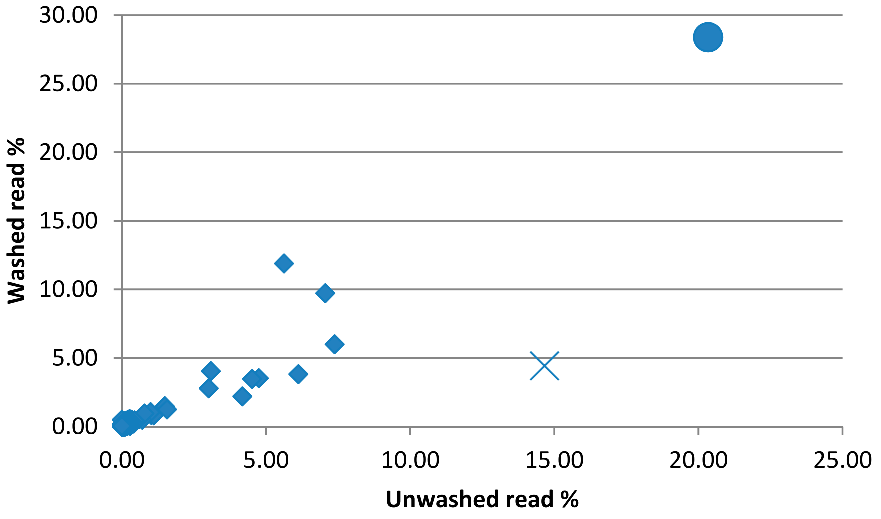

a

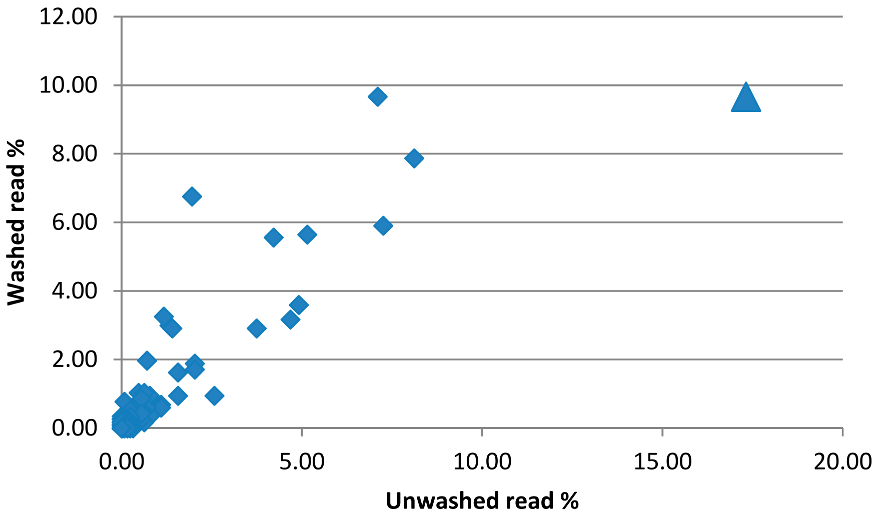

b

Figure S5. The sequence read % for each fungal taxon between unwashed and washed samples of *Fraxinus excelsior* (*H. fraxineus* is marked as X and *V. fraxini* as a circle; a) and the read % for each fungal taxon between unwashed and washed samples of *Sorbus aucuparia* (*Vishniacozyma victoriae* is marked as a triangle; b)

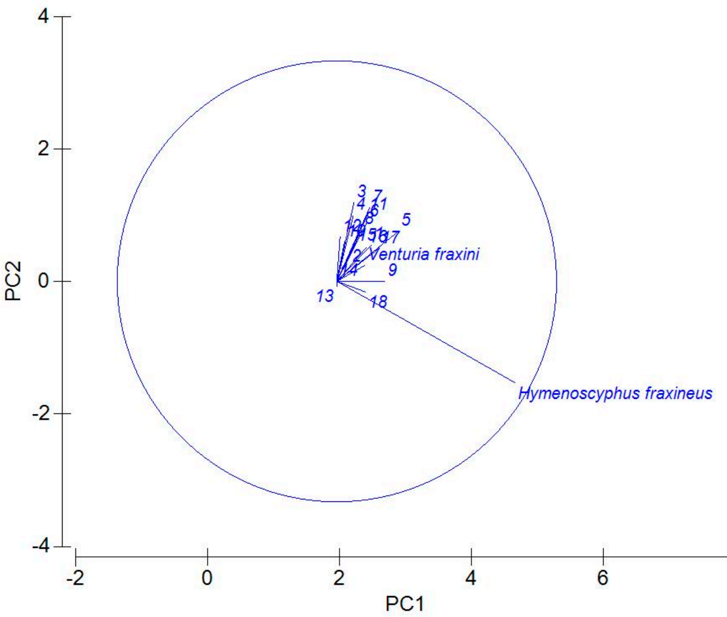

a

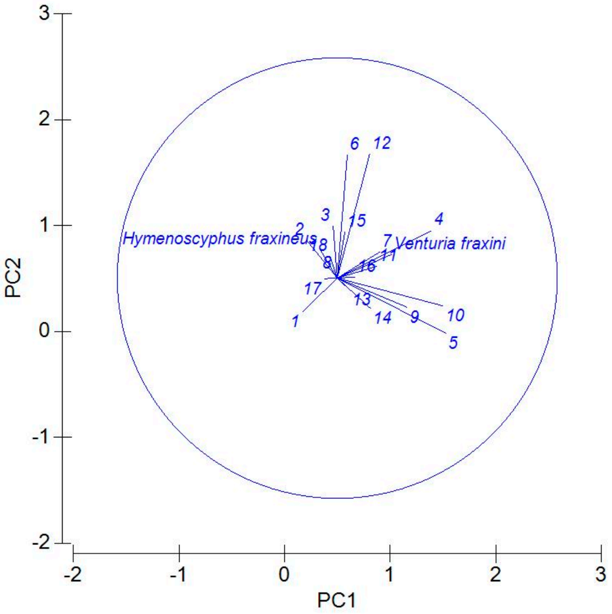

b

Figure S6. PCA plot of read % ratio between unwashed and washed leaflets for 20 most common species on ash (a) and rowan (b), Estonia and Norway data merged

On ash samples 1- *V. victoriae*;2- *P. fraxini*;3- *A. pullulans*;4- *Dioszegia* sp.;5- *C. ramotenellum*;6- *T. carpini*;7- *V. carnescens*;8- *V. heimaeyensis* ;9- *V. foliicola*;10- *V. wieringae* ;11- *T. padi* ;12- *D. crocea* ;13- *N. rosigena*;14- *B. cinerea*;15- *V. flavescens* ;16- *R. aurantiaca*;17- *Taphrina* sp.;18- *E. gracile*

On rowan samples 1- *V. victoriae* , 2- *A. pullulans*, 3- *Dioszegia* sp., 4- *C. ramotenellum*, 5-*T. carpini* 6-*V. carnescens*, 7-*V. heimaeyensis*, 8- *V. foliicola*, 9- *V. wieringae*, 10- *T. padi*, 11- *D. crocea*, 12- *N. rosigena*, 13- *P. flavescens*. 14- *R. aurantiaca*, 15- *A. rosarum*, 16- *Tremmellales* sp., 17- Basidiomycota sp., 18- *P. sessilis*

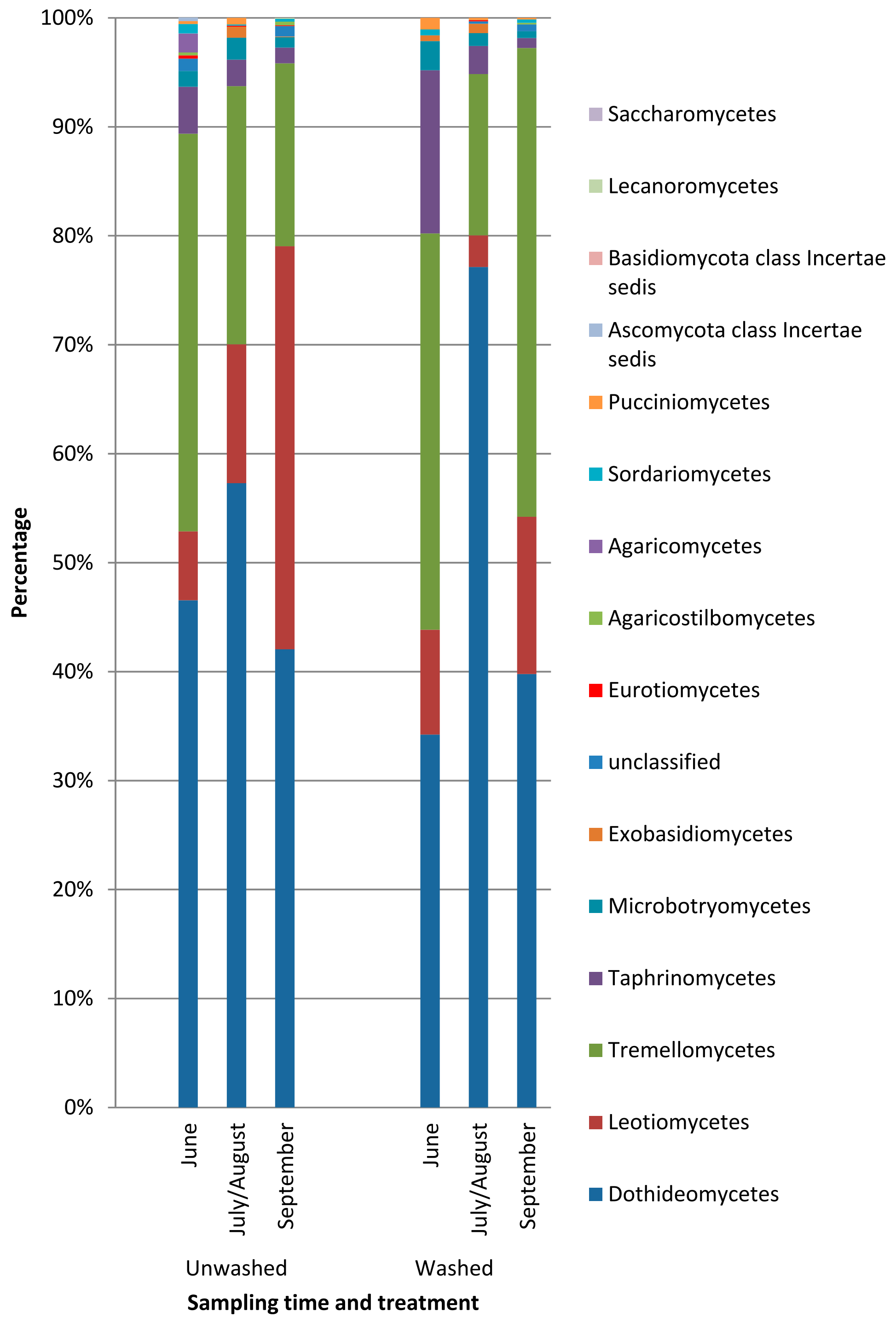
100%
90%
80%
70%
60%
50%
40%
30%
20%
10%
0%
Percentage
June
July/August
September
June
July/August
September
Unwashed
Washed
Sampling time and treatment
Saccharomycetes
Lecanoromycetes
Basidiomycota class Incertae sedis
Ascomycota class Incertae sedis
Pucciniomycetes
Sordariomycetes
Agaricomycetes
Agaricostilbomycetes
Eurotiomycetes
unclassified
Exobasidiomycetes
Microbotryomycetes
Taphrinomycetes
Tremellomycetes
Leotiomycetes
Dothideomycetes

a

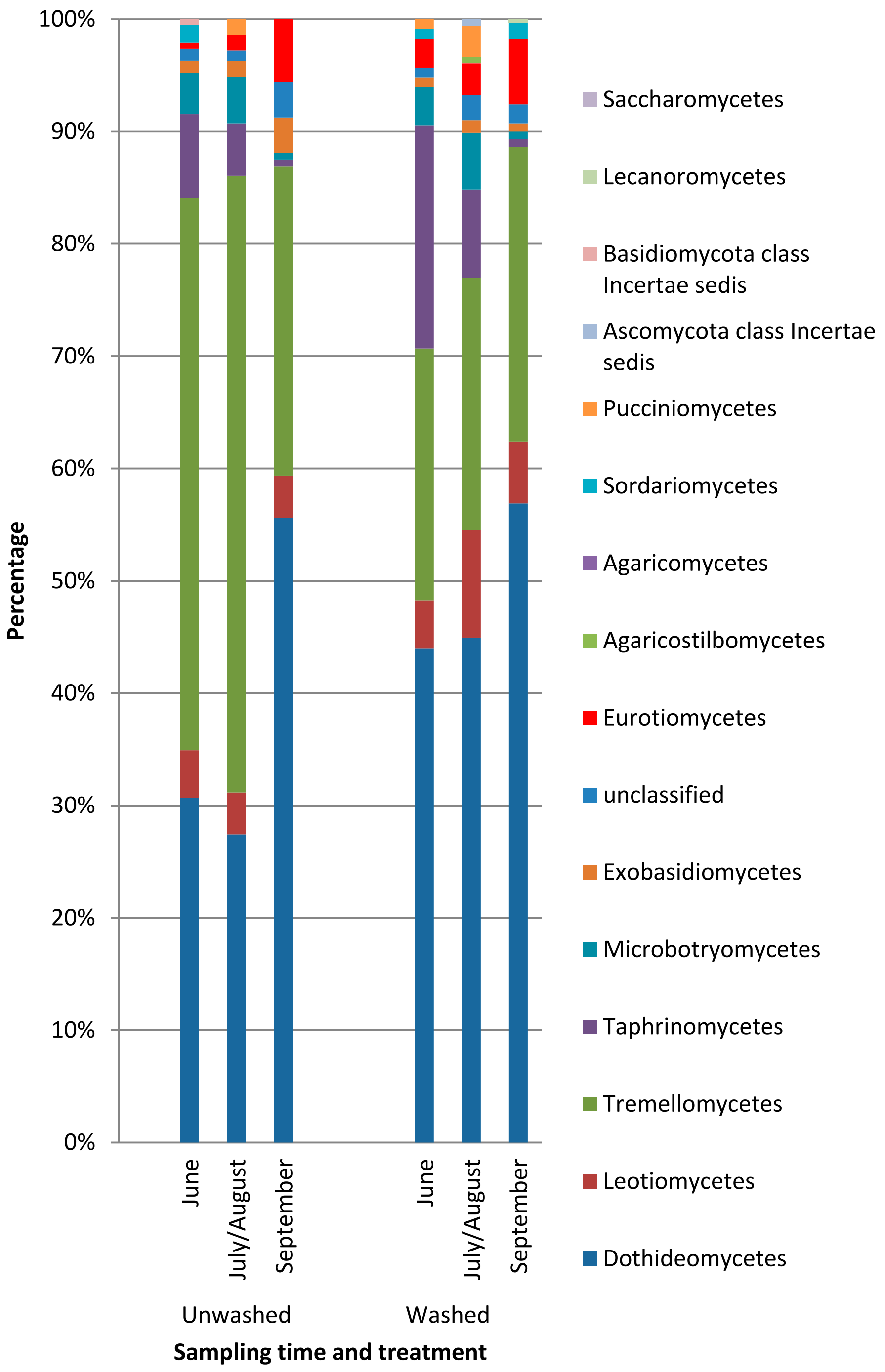

b

Figure S7. Across-season changes in relative proportions of fungal classes across the dataset on ash (a) and rowan (b) in Estonia

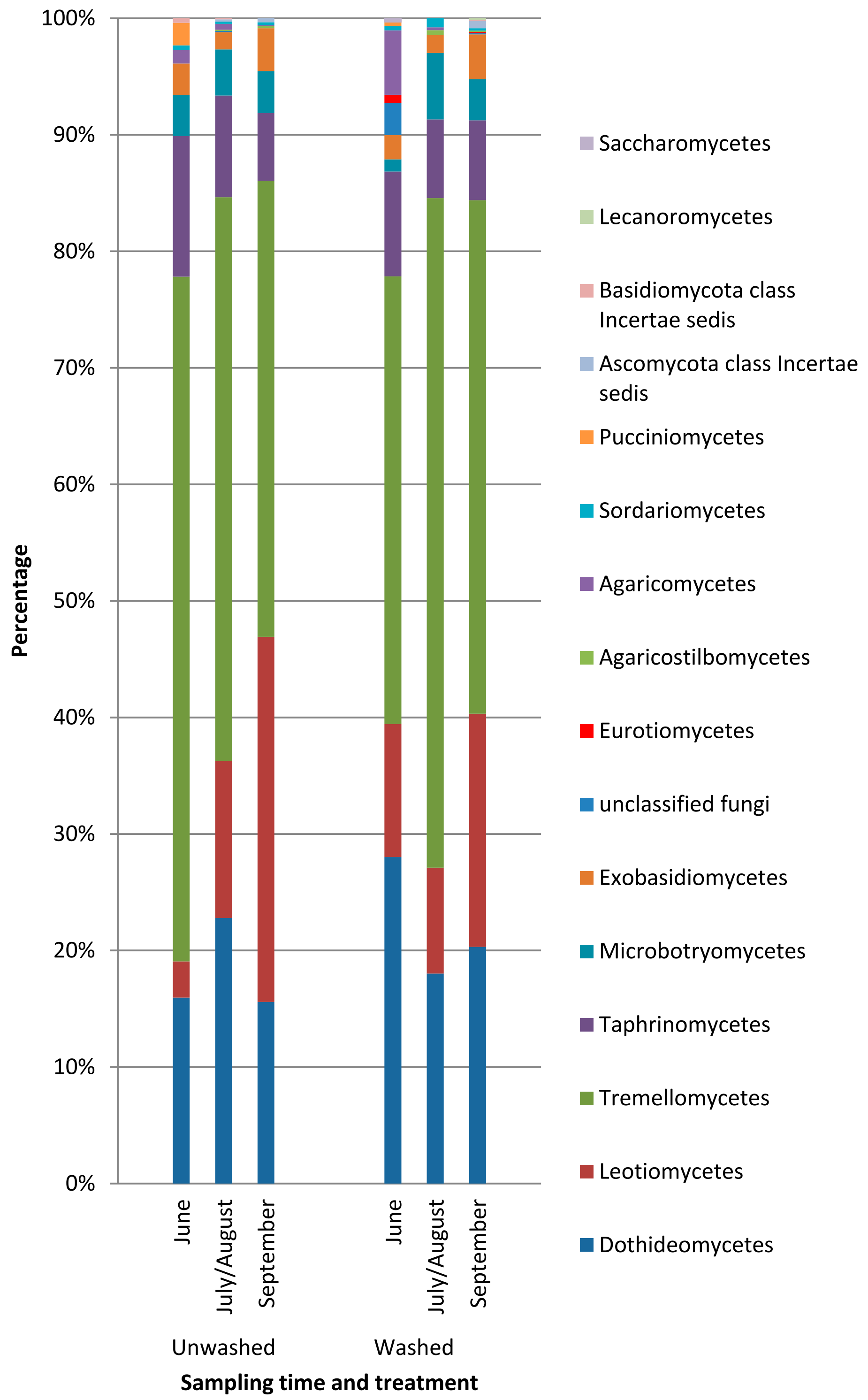
100%
90%
80%
70%
60%
50%
40%
30%
20%
10%
0%
Percentage
June
July/August
September
June
July/August
September
Unwashed
Washed
Sampling time and treatment
Saccharomycetes
Lecanoromycetes
Basidiomycota class Incertae sedis
Ascomycota class Incertae sedis
Pucciniomycetes
Sordariomycetes
Agaricomycetes
Agaricostilbomycetes
Eurotiomycetes
unclassified fungi
Exobasidiomycetes
Microbotryomycetes
Taphrinomycetes
Tremellomycetes
Leotiomycetes
Dothideomycetes

a

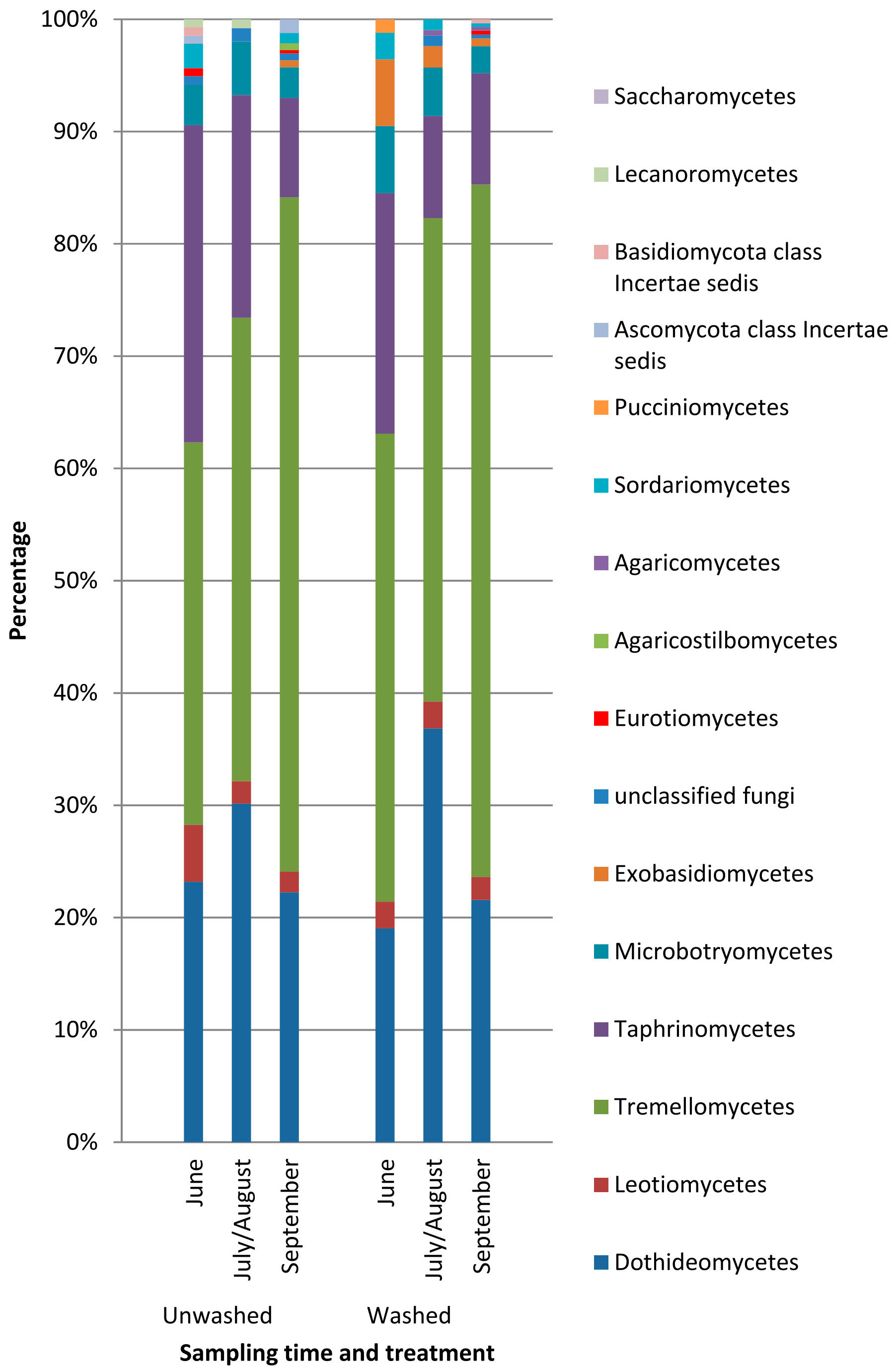

b

Figure S8. Across-season changes in relative proportions of fungal classes across the dataset on ash (a) and rowan (b) in Norway

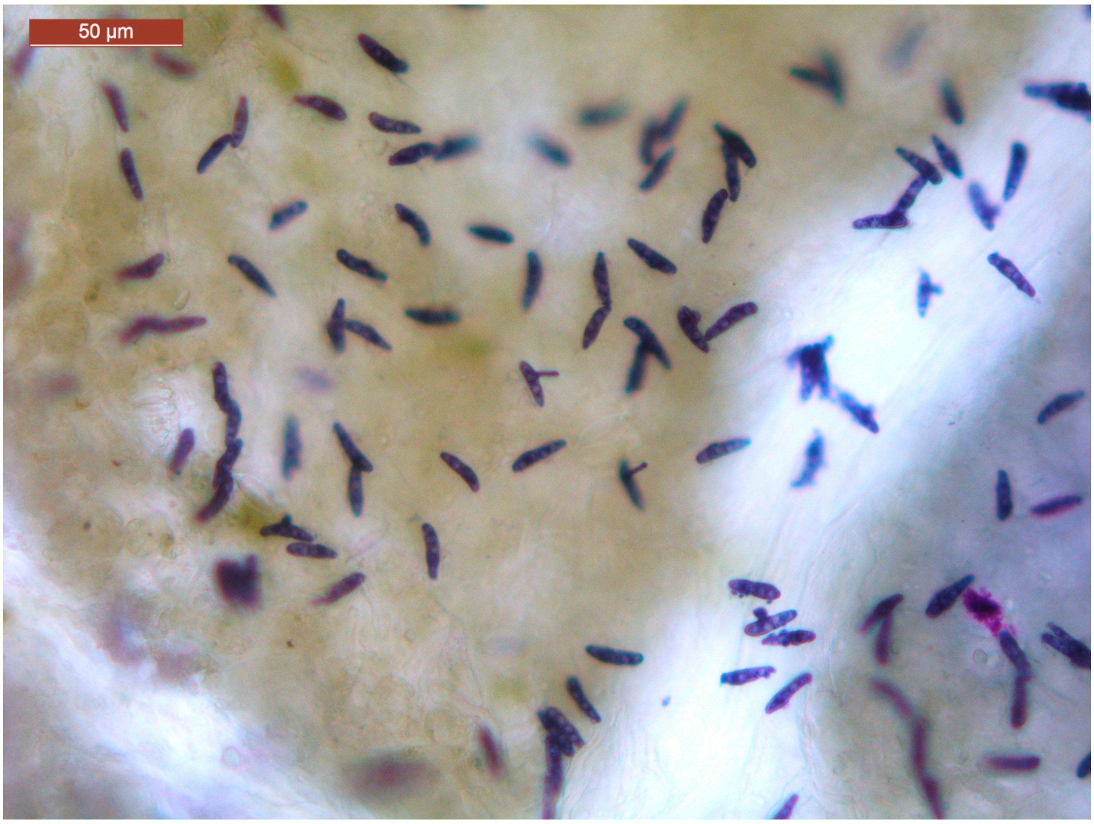

Figure S9. *H. fraxineus* ascospores on detached leaflet of *F. excelsior*. Photo taken 34h after ascospore deposition, note the germ tubes and appressoria-like swellings

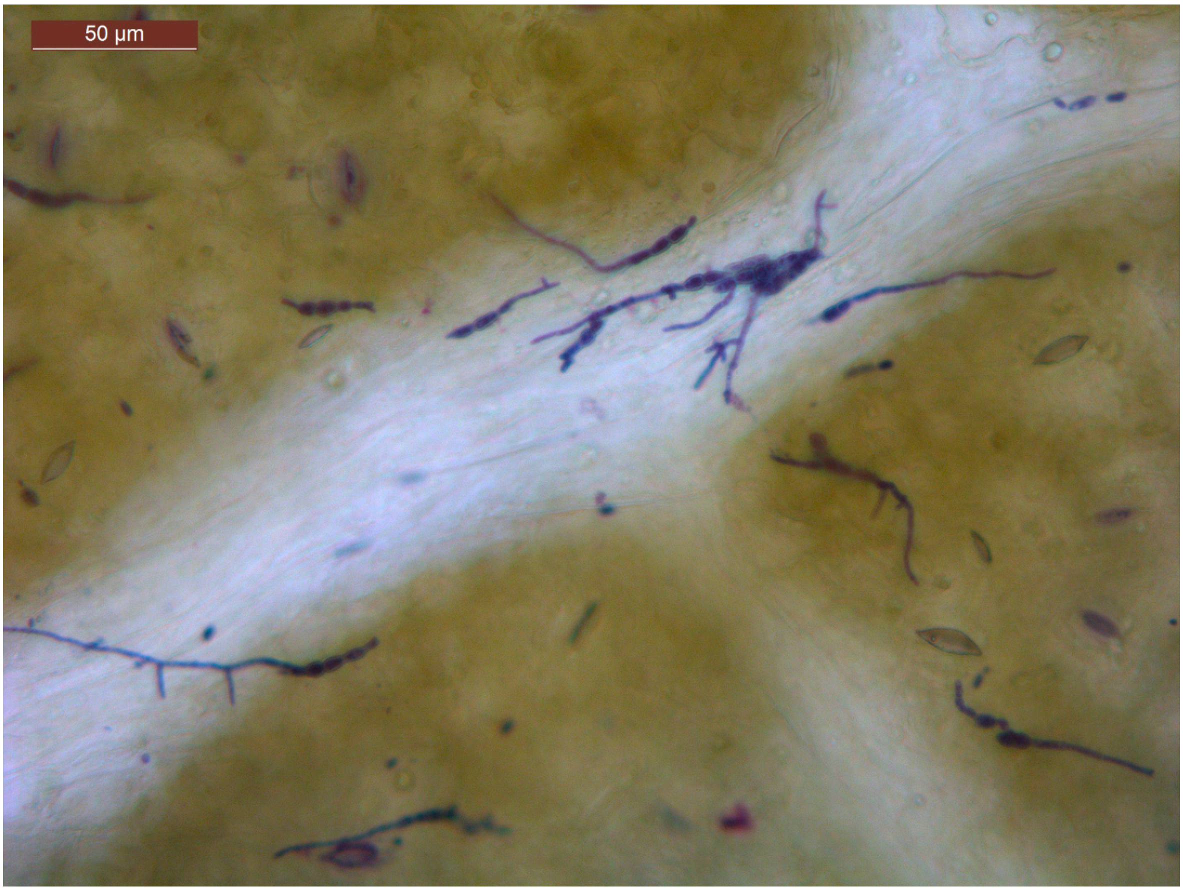

Figure S10. Hyphae of indigenous species growing on veinal regions of ash leaflets and showing intercalary chlamydospores
